# The Hallmarks of Ageing in Human Immunodeficiency Virus Infection and the Impact of Antiretroviral Therapy on Telomeres: A Molecular Perspective

**DOI:** 10.3390/cimb47040273

**Published:** 2025-04-12

**Authors:** Miruna-Maria Apetroaei, Stella Baliou, Petros Ioannou, Persefoni Fragkiadaki, Gabriela Ștefan, Marina Ionela (Ilie) Nedea, George-Traian-Alexandru Burcea-Dragomiroiu, Bruno Ștefan Velescu, Anca Oana Docea, Denisa Ioana Udeanu, Aristidis Tsatsakis, Andreea Letiția Arsene

**Affiliations:** 1 Faculty of Pharmacy, Carol Davila University of Medicine and Pharmacy, 6, Traian Vuia Street, 020956 Bucharest, Romania; miruna-maria.apetroaei@rez.umfcd.ro (M.-M.A.); gabriela.stefan@drd.umfcd.ro (G.Ș.); marina.nedea@umfcd.ro (M.I.N.); george.burcea@umfcd.ro (G.-T.-A.B.-D.); denisa.udeanu@umfcd.ro (D.I.U.); andreea.arsene@umfcd.ro (A.L.A.); 2 Laboratory of Toxicology and Forensic Sciences, Medical School, University of Crete, Voutes, 71003 Heraklion, Greecepersefoni.f@gmail.com (P.F.); aristsatsakis@gmail.com (A.T.); 3 Department of Internal Medicine, University Hospital of Heraklion, 71110 Heraklion, Greece; 4 School of Medicine, University of Crete, 71003 Heraklion, Greece; 5 Department of Toxicology, University of Medicine and Pharmacy of Craiova, 200349 Craiova, Romania; daoana00@gmail.com; 6 Marius Nasta Institute of Pneumophthisiology, 90, Viilor Street, 050159 Bucharest, Romania

**Keywords:** HIV-1, ageing, telomere length, telomere attrition in HIV-1, ageing biomarkers, accelerated ageing, molecular alterations in HIV-1, personalised therapy, pharmacotherapy on telomeres

## Abstract

Ageing is a complex and unavoidable physiological process which, in simple terms, consists of a progressive deterioration in the functionality of cells, tissues and organs, culminating in an increased risk of developing chronic pathologies. Telomeres, the repetitive nucleotide structures at the end of chromosomes, ensure genomic integrity and modulate cellular senescence. The progressive shortening of telomere length with each cell division directly correlates with an increased susceptibility to developing chronic pathologies. However, this shortening, normally physiological and inevitable, can be markedly accelerated in the presence of chronic infections, such as HIV-1 infection, by sustained and continuous activation of the immune system, chronic inflammation, generation of oxidative stress, or direct alterations produced by viral proteins. Thus, in this narrative review, we discuss the 12 hallmarks of ageing in the context of HIV-1 infection, as understanding the molecular changes induced by HIV-1 through these well-established pillars could provide a holistic approach to the management of HIV-positive patients. At the same time, considering that telomeres are at the centre of all these changes, an assessment of the impact of antiretroviral therapy on telomere length is necessary to guide clinical decisions. The ultimate goal of this research is to develop personalised therapies to increase the quality of life and health outcomes of HIV patients.

## 1. Introduction

**Figure 1:**
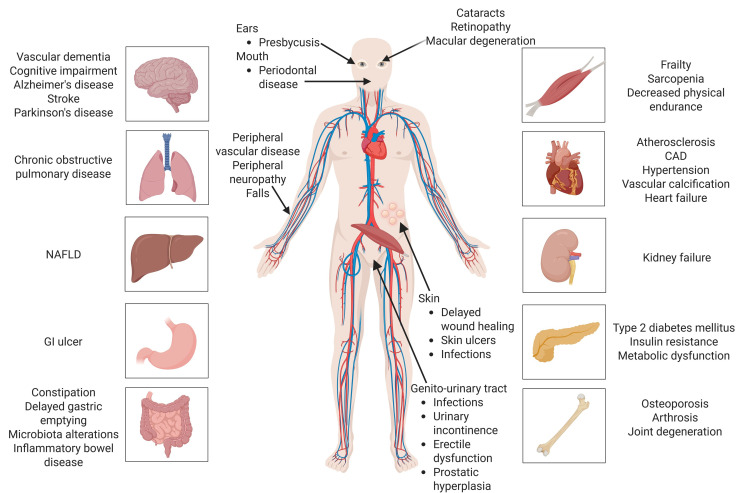
Age-related diseases induced or aggravated by accelerated ageing (Created with BioRender.com). Legend: CAD—coronary artery disease; GI—gastrointestinal; NAFLD—non-alcoholic fatty liver disease.

The ageing process is multidimensional and comprises various interconnected molecular pathways and cellular systems. The biological ageing process is characterised by a progressive decline in the activity of cells and the systemic degradation of several tissues, leading to increased vulnerability to age-related illnesses [1,2]. Ageing is the time-dependent advancement of continuous molecular, metabolic, cellular, and biochemical processes during the lifespan [3,4]. The recognition that individuals age at varying rates gave rise to the notion of biological ageing (BA), often referred to as functional or physiological ageing. Chronological ageing pertains solely to the flow of time, whereas biological ageing concerns the deterioration of function [5]. Normal ageing refers to all observed processes related to ageing where biological age aligns with chronological age. Accelerated ageing (AA) occurs when BA surpasses chronological age, while decelerated ageing is noted when chronological age exceeds BA. AA exhibits similarities with typical ageing; nevertheless, it is distinguished by particular mechanisms [6]. Figure 1 provides an illustration of the most prevalent age-related illnesses, whereby individuals with elevated levels of AA become progressively vulnerable to them.

Research on telomeres, first identified in 1975, has suggested that these activities revolve around the caps that protect the ends of our chromosomes. Somatic cell replicative senescence could be caused by telomere shortening, according to Alexey Olovnikov’s telomere theory of ageing. This happens as DNA polymerase becomes incapable of copying the last DNA sequence, which causes chromosomes to shrink with each cell division. Based on experimental evidence, chromosome ends are estimated to be reduced by 50 pairs of bases in each human cell division and by 2–5 kilobases per generation [7]. The most important role of telomeres is to ensure that the ends of chromosomes are correctly identified when DNA breaks and do not fuse with other chromosomes [8]. TTAGGG is a sequence of double-stranded tandem repeats found in telomere DNA, which is followed by single-stranded overhangs that are rich in G at the 3′ end. The accepted view holds that telomere DNA takes on the shape of a T-loop, with the telomere end folding around itself with the 3′ G strand overhang invading the double-stranded DNA (also known as a D-loop) [9]. However, it is the G-rich sequence that makes telomeres so vulnerable to oxidative degradation. Moreover, reactive oxygen species (ROS) might cause telomere shortening by inducing single-strand breaks at the ends of DNA strands [10].

Senescent cells, which increase in aged organisms, are believed to have a role in physiological malfunction [11]. Cellular senescence significantly impacts the preservation of normal tissue homeostasis and pathological circumstances. It is the progressive deterioration of cellular proliferation, differentiation capacity, and physiological function over time that serves as a primary causal component in the ageing process [12]. One known cause of cellular senescence and ageing is telomere shortening. Accelerated telomere shortening predisposes individuals to chronic pathologies [13], including cardiovascular disease [14], neurodegenerative disorders [15], cancer [16] and so on. The persistent cellular stress and systemic inflammation linked to these conditions further exacerbate telomere attrition, creating a bidirectional cycle that contributes to the onset and progression of comorbidities [17].

## 2. Current Perspectives on HIV Infection

The human immunodeficiency virus (HIV) belongs to the genus *Lentivirus* within the *Retroviridae* family, subfamily *Orthoretrovirinae*. HIV is categorised into two forms, HIV-1 and HIV-2, based on genetic traits and variations in viral antigens. Epidemiological and phylogenetic investigations suggest that HIV spread into the general population of humans between 1920 and 1940 [18].

Eleven genes make up HIV-1; three are structural (*gag*, *pol*, and *env*), two are regulatory (*tat*, *rev*), and the remaining four are accessory (*vif*, *vpr*, *vpu*, *nef*) [19]. HIV enters cells via the binding of HIV glycoproteins to specific cell membrane receptors, particularly through the interaction of the HIV envelope glycoprotein gp120 with the primary CD4 receptor and its coreceptors, CCR5 and CXCR4. The simultaneous binding induces conformational changes that enable the fusion fragments (gp41) to integrate into the cell and facilitate membrane merging [20]. Employing reverse transcriptase and RNA-dependent DNA polymerase, HIV-1 transforms its RNA into DNA. Subsequently, it relies on integrase to integrate its viral DNA with the host cell’s DNA [21]. This leads to the transcription and translation of mRNA by the host’s cellular machinery, resulting in the production of either replication-competent or incompetent (pro)viruses. The proviral DNA is integrated into the host DNA and persists even with prolonged exposure to antiretroviral therapy (ART), establishing what is referred to as the viral reservoir [20]. ART targets various stages of the HIV life cycle to inhibit viral replication. A diverse range of antiretroviral drug classes has progressed to clinical application for HIV treatment. These include pre-attachment, post-attachment, and fusion inhibitors; capsid inhibitors; non-nucleoside reverse-transcriptase inhibitors (NNRTIs); nucleoside and nucleotide reverse-transcriptase inhibitors; integrase strand transfer inhibitors (NRTIs); and HIV protease inhibitors (PIs). Optimal pharmacotherapy involves the administration of multiple antiretroviral drugs, usually three, from at least two distinct classes to effectively suppress viral replication and minimise the risk of drug-resistant virus development in the patient. This treatment aims to lower plasma viral loads to undetectable levels and inhibit the progression of HIV infection to AIDS. While ART effectively inhibits the replicating virus, it fails to target stable reservoirs of HIV [22,23]. Lifelong treatment is mandatory, as the virus remains in latent reservoirs—cells in which HIV integrates into the host genome—eluding immune responses and standard pharmacotherapy. Latent reservoirs represent a group of enduring cells, mostly resting memory CD4+ T lymphocytes, that contain integrated yet transcriptionally inactive HIV provirus. The virus is able to evade immune detection and viral reproduction by remaining in a dormant state, which means that these reservoirs continue to exist even after ART is initiated. Cellular stimulation can trigger the reactivation of the latent virus, resulting in viral rebound if antiretroviral therapy is discontinued. Latent reservoirs provide a significant obstacle to research aimed at discovering a treatment for HIV [24].

Annually, with the support of the Joint United Nations Programme on HIV/AIDS (UNAIDS), national teams worldwide generate estimates that outline the status of their respective HIV epidemics. In 2023, forecasts regarding HIV were accessible for 174 countries, representing 99% of the world’s population, with teams from 150 of these countries actively participating in this effort [25]. According to UNAIDS, in 2023, there were 39.9 million individuals worldwide living with HIV, and 630,000 individuals died of AIDS-related ailments during the same year [26].

Combination ART has reduced HIV-related morbidity and mortality. HIV therapy focuses on long-term virologic suppression with ART. With the availability of once-daily dosage and fixed-dose combinations, modern ART regimens have become more potent, less toxic, and simpler. Thus, HIV-infected people who receive proper care can expect to live four decades longer, which is comparable to the normal population [27].

The contemporary framework of HIV care extends beyond the mere suppression of HIV-RNA plasma viral load and the recovery of CD4+ T cells, incorporating both pharmacological and non-pharmacological factors, whether general or individualised, that contribute to the sustained success of ART. Furthermore, the conventional notion of virological suppression has progressed due to emerging information regarding the clinical importance of plasma low-level HIV-RNA and the availability of assays with reduced thresholds for viral RNA detection in numerous countries globally [28]. More than 24 million people received ART medication in June 2019. However, increased availability of ART has been linked to HIV drug resistance, which is expected to increase mortality, program expense, and HIV incidence [29,30]. All primary classes of antiretrovirals face cross-drug resistance. HIV medication resistance among ART-naive people is rising exponentially, making it challenging to end the HIV-1 pandemic by 2030. A total of 10% of HIV-positive persons starting therapy are non-nucleoside reverse transcriptase inhibitors (NNRTI)-resistant. Patients on ART are threefold more likely to be resistant [23].

To expedite advancements in the battle against HIV, various novel approaches and prospective paths are being explored. These pertain to the advancement and assessment of novel preventive technologies, including HIV vaccinations and long-acting pharmaceuticals. The combined effort of HIV services with other health initiatives, including services related to reproductive and sexual health and primary healthcare, augments accessibility and promotes health outcomes. Discrimination and stereotyping, together with other inequalities in socioeconomic status and exclusion, are significant obstacles to eradicating AIDS [31,32]. Fostemsavir was the first FDA-approved attachment inhibitor for adults with multidrug-resistant HIV-1 who have undergone extensive treatment. It serves as a prodrug for temsavir, the active compound that obstructs the conformational alterations of gp120 essential for CD4 binding, thereby hindering viral entry into target cells [33]. Ibalizumab was the first monoclonal antibody authorised for treating HIV-1 infection. It functions as a long-acting post-attachment inhibitor by binding to the extracellular CD4 domain, which induces conformational changes in the CD4 T cell receptor–gp120 complex, thereby obstructing HIV entry [34]. Leronlimab is a humanised IgG4 monoclonal antibody functioning as an HIV-1 CCR5 antagonist. It binds to the hydrophilic extracellular domains of CCR5, thereby inhibiting HIV-1 viral entry through competitive inhibition [35]. Since October 2024, only one long-acting injectable ART regimen—cabotegravir plus rilpivirine—has been available for monthly or every other month administration. Other approaches include a weekly islatravir–lenacapavir combination in clinical trials [36,37].

## 3. The Hallmarks of Ageing in HIV Infection

An integrative view was needed to understand the quasi-universal ageing process, which is complex and involves many elements that interact. In 2013, López-Otín et al. defined ageing as having nine hallmarks categorised as primary, antagonistic, or integrative. They also established three criteria for a biological process to be considered a hallmark of ageing: it must appear during physiological ageing, accelerate ageing when exacerbated empirically, and slow ageing when alleviated empirically, improving healthy longevity [38]. In 2023, Lopez-Otin et al. reorganised these hallmarks and included three more: impaired macroautophagy, chronic inflammation, and dysbiosis [39].

The abovementioned characteristics are not autonomous but hierarchically interrelated. The principal hallmarks of ageing are those that commence the process, as the damage they inflict accumulates over time; the antagonistic hallmarks initially serve beneficial functions, but eventually, partly due to the damage caused by the primary hallmarks, they become harmful; finally, the integrative hallmarks emerge when the damage inflicted by the other categories exceeds the capacity of homeostatic mechanisms [40]. Importantly, they can serve as a model for future studies on the molecular aspects of ageing, the connections between different ageing phenotypes, and potential intervention points in the ageing process in order to increase treatment outcomes in vulnerable patient populations [41].

The presence of HIV infection introduces additional complexity to the heterogeneous process of ageing. Current evidence indicates that various biological ageing mechanisms affect individuals living with HIV. While many hallmarks of ageing are likely intensified in the context of HIV, current research is yielding new insights into the combined effects these preserved pathways might have on age-related disease processes [42]. HIV-positive patients have a much greater rate of age-related comorbidities than HIV-negative adults across cohorts. Attempts to shed light on this disproportionately associated burden in HIV-positive adults have suggested that HIV infection promotes or exacerbates ageing irrespective of established risk factors without linking ageing mechanisms to disease vulnerability. Due to the predominance of age-related illnesses, most healthcare spending happens near the end of life, even if evidence-based therapies can extend longevity. Improving healthspan (the period of an individual’s life throughout which they are typically in good health) is thus expected to have a more substantial impact on a population’s well-being and economic indices [43].

The ultimate goal of this holistic approach is to identify new therapeutic targets to improve patient outcomes and design appropriate personalised therapeutic plans in order to enhance the quality of life of HIV/AIDS patients. A first step in this direction would be the routine assessment of biomarkers associated with the ageing hallmarks. Incorporating these biomarkers into clinical practice could provide an innovative method, allowing healthcare professionals to shift from the conventional emphasis on HIV viral load and CD4 counts towards a more holistic comprehension of the patient’s biological age and susceptibility to comorbidities. Through routine monitoring of these biomarkers, clinicians can detect early indicators of biological ageing, categorise patients according to the risk of age-related comorbidities, and personalise therapies that reduce these risks. The ultimate goal of this approach is to identify new therapeutic targets to improve patient outcomes and design appropriate personalised therapeutic plans in order to enhance the quality of life of HIV/AIDS patients. Here, we discuss the mechanisms by which HIV infection promotes accelerated ageing and the onset of comorbidities in this patient population, intending to provide a holistic understanding of the molecular processes involved that could lead to new personalised therapeutic approaches.

### 3.1. Primary Hallmarks of Ageing

The primary pillars associated with ageing are innately harmful and consist of five fundamental indicators: genomic instability, telomere attrition, epigenetic changes, loss of proteostasis, and disabled macro-autophagy [40].

#### 3.1.1. Genomic Instability

Genomic instability covers a spectrum of DNA alterations, including point mutations, deletions, insertions, chromosomal rearrangements, and alterations in chromosome number. These modifications result in irreversible changes to the informational content of the genome [44]. Cells are required to thoroughly secure the genetic information encoded within their DNA and reliably transmit that information to subsequent generations. Nevertheless, DNA is not a passive molecule; thus, various types of DNA lesions must be identified, communicated, and rectified by the DNA damage response (DDR) apparatus to prevent the genomic instability associated with advancing age, neurodegeneration, and malignant events [45]. DDR is a signalling cascade responsible for detecting and responding to irregularities in nucleic acids. Nevertheless, besides preserving genomic integrity, DDR is adeptly positioned to modulate viral infections, as viruses are fundamentally perceived by the cell as anomalous nucleic acids. In this context, the relationship between viral replication and the DDR has been identified in two principal capacities: viruses manipulate the DDR proteins and pathways necessary for their replication, and interactions involving the DDR and the immune system’s natural defences directed against viral infections exist [46].

The DDR can be triggered by the presence of viral nucleic acids, including reverse-transcribed viral DNA during the integration of retroviruses such as HIV-1, or by abnormal DNA structures formed during the replication of DNA viruses. Moreover, viral proteins derived from DNA and RNA can elicit the DDR by facilitating erroneous entry into the S phase, directly altering cellular DDR components, or inadvertently interacting with host DNA. DDR may possess antiviral properties; however, viruses frequently necessitate the proximal activation of DDR repair and rearrangement elements to promote their replication and suppress downstream DDR signalling to guarantee cellular survival [47].

In particular, Vpr induces replication fork collapse, leading to both single-strand and double-strand DNA breaks. In addition, Vpr and the other HIV-1 proteins (Tat and Vif) might damage DNA by inhibiting critical DNA repair pathways [48]. This genotoxicity may, at least in part, be explained by the cellular stress and genomic instability triggered during the integration of reverse-transcribed HIV-1 DNA into the host genome [49]. Consequently, Vpr can trigger the production of micronuclei and other chromosomal abnormalities. It is noteworthy that Tat, Vif, and Vpr facilitate cellular arrest, which may enhance viral replication. Furthermore, latent HIV-infected cells exhibit increased vulnerability to DNA damage [48]. In the same direction, exposure to Nef resulted in substantial elevations of activation-induced cytidine deaminase (AID) and c-MYC, both of which contribute to genomic instability [50]. AID induces mutations and DNA double-strand breaks by deaminating cytosines in DNA, a process normally restricted to immunoglobulin gene diversification [51]. However, in the context of HIV infection, Nef-driven AID expression becomes aberrant, promoting off-target mutations and DNA damage that may predispose cells to malignant transformation. Therefore, HIV proteins may help account for the increased incidence of HIV-associated lymphomas, including Burkitt lymphoma, among the HIV-infected population [50].

Moreover, genome function can be disrupted, genome instability can be induced, and mutation can occur as a result of reactive oxygen species’ base-modifying capabilities. Both initial oxidative DNA damage and its subsequent repair contribute to these alterations. As a result, the cell must decide whether to fix a broken base at a particular chromosomal position or leave it unrepaired [52]. A plethora of evidence indicates that HIV-1 infection induces significant oxidative stress in both laboratory models and in vivo contexts by disrupting oxidative stress pathways, leading to increased ROS generation and mitochondrial dysfunction. Consequently, HIV patients display various indicators of oxidative stress, including DNA damage. The augmentation of ROS generation is facilitated by gp120, Tat, Nef, and the reverse transcriptase. Tat triggers oxidative stress through both direct and indirect pathways via multiple distinct mechanisms. The first pertains to NADPH oxidases, the second to spermine oxidase, an enzyme implicated in the metabolism of biogenic polyamines, and the third to mitochondrial malfunction [53]. Louboutin et al. demonstrated that both direct infusion and cellular production of gp120 result in the rupture of the blood-brain barrier by elevating matrix metalloproteinases and diminishing vascular proteins at tight junctions through pathways involving the production of reactive oxygen species and oxidative damage [54]. While the exact processes are still poorly understood, Isaguliants et al. demonstrated that expressing HIV reverse transcriptase in human embryonic kidney cells increases ROS generation and induces oxidative stress responses [53]. Moreover, another study utilising a murine model highlighted that myristoylated Nef increases levels of inducible nitric oxide synthase in microglia cells, which may have neurotoxic consequences on other cell types, including neurones [55].

**Figure 2:**
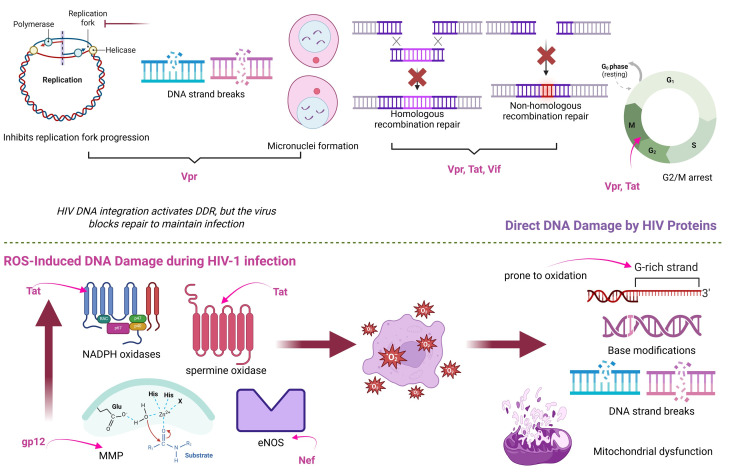
HIV-1-induced DNA damage (Created with BioRender.com). Legend: HIV—human immunodeficiency virus; ROS—reactive oxygen species; Vpr—viral protein R; Tat—transactivator of transcription; Vif—viral infectivity factor; gp120—glycoprotein 120; Nef—negative regulatory factor; G-rich—guanine-rich; eNOS—endothelial nitric oxide synthase; MMP—matrix metalloproteinases; NADPH oxidases—nicotinamide adenine dinucleotide phosphate oxidase; DDR—DNA damage response.

Moreover, an accumulation of reactive oxygen species (ROS) can result in DNA damage, evidenced by the presence of micronuclei and nuclear aberrations. Gutiérrez-Sevilla et al. noted that individuals who are HIV-positive exhibited a greater prevalence of nuclear buds and binucleated cells compared to their HIV-negative counterparts, despite an equivalent count of micronucleated cells in both cohorts [56]. Micronuclei are generated from chromosomes and chromosomal fragments that lag during anaphase and remain outside the daughter nuclei in telophase. They may also originate from fractured anaphase bridges. Nuclear buds, micronucleus-like structures linked to the nucleus by a slender nucleoplasmic connection, are hypothesised to form in a manner equivalent to micronuclei during nuclear division or in the S-phase, perhaps serving as a precursor to the extrusion of excess DNA, which may lead to the formation of micronuclei [57]. Figure 2 summarises the most important molecular mechanisms induced during HIV-1 infection contributing to DNA damage.

#### 3.1.2. Telomere Attrition

Since HIV infection hastens biological ageing, leading to cellular damage, programmed cell death, or senescence, it is logical that patients with HIV infection exhibit an accelerated rate of telomere shortening [42]. Crucial characteristics related to telomere length are viral load and CD4 T-cell count. In this manner, the ageing rate is accelerated by one decade upon exposure to HIV infection [58]. For example, telomere length is reduced by 13% within three months of HIV seroconversion [59]. In a case-control study, South African adults with HIV presented shorter telomeres than those who were HIV-seronegative [60].

Besides, a recent CARMA cohort study has illustrated that people living with HIV present lower telomere length values compared to healthy individuals after telomere length quantification with monochrome multiplex qPCR [61]. In HIV patients, the accelerated telomere shortening is linked to immunological ageing due to the increased prevalence of other infections [61]. In a group of HIV-positive women, telomere shortening was observed, and this positive relationship was attributed to the molecular mechanisms of HIV infection [58]. The rate of telomere shortening seemed to be aggravated in HIV-infected women who also carried active hepatitis C virus infection [58]. Telomere length shortening is exacerbated separately by HIV and parasite infections [62]. Coinfection with HIV and parasites can also promote telomere length shortening. However, this is an unexplored area with no published studies [62]. Consistent with this, the South African population co-infected with HIV and helminths has a more significant telomere length reduction than those who are infected with either HIV or helminths alone [63]. The mean relative telomere length was observed to be highest in the uninfected control group. In contrast, those values were lower in the HIV-infected and co-infected group with HIV and helminth, following the same trend. Notably, the group with helminth infections had the lowest relative telomere length [63].

Regarding HIV-infected patients, it was shown that telomere length declines in a genetically dependent manner. A significant telomere shortening was observed during untreated chronic HIV infection, while no telomere length change was detected during suppressive ART, suggesting stabilisation rather than recovery. However, telomere dynamics during ART did not differ significantly from those observed in HIV-negative controls [64]. In particular, this longitudinal study highlights the precise reduction of telomere length values in an individualised manner related to single-nucleotide polymorphisms in the genome of every individual [64].

Telomeres of HIV-infected people aged below 35 years were reported to be longer than those of HIV-infected people above 50 years, confirming the telomere shortening in an age-dependent manner during HIV infection. Interestingly, a trend toward longer telomeres was observed in young HIV-infected individuals compared to age-matched healthy controls. However, this difference was not statistically significant, indicating that telomere lengths were comparable. Despite the mounting evidence supporting the inverse relationship of telomere length with HIV infection, antiretroviral therapy seems to exacerbate this. Interestingly, the telomere shortening rate was accelerated in patients receiving antiretroviral therapy [65].

Non-AIDS comorbidities affect people living with HIV (PLWH) more frequently than the general population, largely due to persistent inflammation occurring during HIV infection. The onset of age-related complications is present even in HIV-infected individuals who have viral suppression and a recovered CD4 T-cell population. Furthermore, HIV infection is consistently associated with chronic immune activation and persistent systemic inflammation [66]. Telomere length has been proposed as a potential biomarker to identify individuals at higher risk of neurocognitive decline in this population [67]. Accordingly, the Swiss HIV Cohort Study has shown that telomere length, in combination with a polygenic risk score, may also help identify individuals at risk for coronary artery disease [68].

Recent findings indicate that newborns of HIV-infected mothers, even when uninfected themselves, exhibit significantly shorter leukocyte telomeres compared to those born to HIV-negative mothers. This telomere shortening appears independent of maternal viral load at delivery and is likely influenced by in utero exposure to ART and oxidative stress. These results suggest that maternal HIV infection and its treatment may impact fetal telomere biology, potentially affecting the long-term health of exposed but uninfected children [69].

Apart from using telomere length as a marker of biological ageing, the DNA methylation estimator of telomere length (DNAmTL) has been suggested as a surrogate of telomere length after a comparative analysis of DNA methylation and telomere length, which share similar age-related changes [70,71]. In particular, DNA methylation (DNAm) at cytosine-phosphate-guanine dinucleotides (CpGs) islands and evaluation of telomere length using different methods presented similar trends, providing insights into the accurate estimation of the biological ageing [70,72,73,74]. Additionally, research has demonstrated that DNAmTL is superior to the telomere length method, providing accurate information about increased all-cause mortality risk [70,71]. In this context, it was revealed that the average DNAmTL values of HIV-positive individuals were lower than those of healthy individuals [71].

#### 3.1.3. Epigenetic Alterations

Epigenetics originally referred to the complex genome-environment interactions that affect higher organism growth and differentiation. Today, this word refers to heritable changes without DNA sequence changes. Instead, epigenetic modifications, including DNA methylation, histone modification, and chromatin conformation, change DNA accessibility and structure, influencing gene expression [75].

The DNA methylation of host gene sequences in reaction to HIV-1 infection has been associated with processes related to viral latency, HIV-1 gene transcription, and viral replication [76]. As an epigenetic mark, DNA methylation controls whether genes are activated or silenced during transcription. DNA methyltransferases (DNMTs) expression and activity are enhanced when CD4+ T cells are infected with HIV-1. In lymphoid cell lines, hypermethylation and decreased expression of the tumour suppressor gene, p16^INK4A^, are outcomes of acute infection with either wild-type or integration-defective HIV-1 [77]. DNMTs include DNMT1, DNMT3a, and DNMT3b, which are responsible for establishing DNA methylation. DNMT1 controls methylation patterns after DNA replication, and DNMT3a and DNMT3b control methylation during DNA synthesis. As a result, DNA methylation levels across cells tend to shift when DNMT expression is altered, such as the upregulation of DNMT expression in CD4+ T cells infected with HIV. The virus promotes DNMT1 expression in a tissue-agnostic way, and upregulation of the viral genes, *nef*, *tat*, and *rev*, activates the DNMT1 promoter [78].

Using CD4+ T cells from 12 healthy donors, Jorge Meneses Nunes et al. examined gene expression, total DNA methylation, and HIV-1 replication dynamics for 36 h following infection. During HIV-1 replication in CD4+ T cells, the epigenetic targets aurora kinase B (AURKB), AURKC, and DNMT3B, as well as the total DNA methylation profile, are regulated. This can be influenced by the cell’s activation state at infection. HIV-1-infected cells showed genomic, transcriptomic, and proteomic changes. The data indicate that DNA methylation and differential expression of AURKB, AURKC, and DNMT3B additionally play a role in HIV-1 replication regulation in CD4+ T cells and viral latency, particularly in resting cells. Many elements of HIV-1 gene activity in active and resting cells appear hostile. The induced state also affects epigenetic regulation in HIV-1-infected cells, showing the virus’s adaptability to varied cellular settings [79].

Moreover, FOXP3 methylation positively correlated with blood CD4+ levels. DNMT1, DMAP1, METTL7B, and METTL10 were considerably down-regulated in HIV-infected patients relative to controls and correlated positively with FOXP3 promoter methylation. A decreased degree of FOXP3 promoter methylation was substantially related to alterations in FOXP3 gene and protein expression and an elevated level of T reg, which may be induced by HIV infection [80]. In the same direction, Pion et al. found that regulatory T cells’ expression of FOXP3 is downregulated by HIV-1 infection. This is linked to an increase in DNMT3B expression and increased methylation of 5′—C—phosphate—G—3′ (CpG) sites in the FOXP3 locus [81].

However, scepticism surrounds DNA methylation’s function in HIV-1 latency. In HIV-positive patients who developed cancer as a co-morbidity, DNMT levels tend to drop. Inducing DNMT3 expression and consequent DNA hypermethylation is possible through the viral Tat protein [78].

Furthermore, the host epigenetic regulatory apparatus modulates the proviral genome. By influencing the chromatin structure in close proximity to the viral promoter, which is situated in the 5′ LTR sequence, cellular epigenetic regulators modulate HIV latency and reactivation [82]. The HIV LTR acts as a potent transcriptional enhancer, integrating binding sites for various host transcription factors, including NF-κB, Sp1, and AP-1. This enables rapid and high-level expression of viral genes upon cellular activation. The strong enhancer activity of the LTR plays a central role in both acute viral replication and the potential for reactivation from latency, particularly in response to inflammatory stimuli and stress pathways [83,84]. Subsequently, the gene expression pattern and epigenotype of the host cells are influenced by specific HIV proteins [82]. Recent findings indicate that the histone chaperone CAF-1 interacts with the provirus LTR. CAF-1 may also play a role in HIV-1 latency through the formation of phase-separated nuclear bodies, in addition to its recruitment of chromatin modifiers [85]. Besides, viral Tat interacts with a stem-loop configuration at the 5′ terminus of viral mRNA, decreasing this inhibition by facilitating a reorganisation of the nucleosome structure situated downstream from the transcription-initiation site. Tat executes this function by enlisting the transcriptional coactivator p300 and its closely related counterpart, the CREB-binding protein, to the viral LTR, both of which possess histone acetyltransferase activity [86]. HIV gene regulation involves chromatin nucleosomal histone changes that change transcription factor recruitment to viral DNA. In a small number of latently infected cells, changes in histone structure near the HIV LTR region help maintain the virus in a silent state. Class I histone deacetylases (HDACs) 1, 2, and 3 establish and maintain this latency by removing acetyl groups from histones, leading to tighter chromatin and reduced gene expression. These HDACs are active in resting CD4+ T cells and can suppress HIV expression, but their inhibition with specific drugs can reactivate the virus ex vivo. In contrast, inhibiting class II HDACs (such as HDACs 4, 5, 7, and 9) does not lead to viral reactivation [87]. For HIV to become active after integration into the host genome, the LTR region must undergo chromatin remodeling and histone acetylation, allowing access to transcription factors like NF-κB. Persistent silencing of the provirus is mainly due to these local chromatin effects [88].

An investigation by Espíndola et al. linked immunological failure in monocytes from ART-naive and ART-treated HIV-positive patients to epigenetic alterations. HIV-positive monocytes had impaired phagocytosis, cytokines, and ROS generation after *M. tuberculosis* infection in vitro. HIV infection affected epigenetic enzyme expression, which was particularly pronounced in patients with significant amounts of soluble CD163, a plasma biomarker of HIV infection progression. Histone acetyltransferase 1 was identified as the most effective epigenetic biomarker for HIV-soluble CD163 high patients. HIV impairs monocyte effector capabilities and is related to epigenetic modifications that could be employed as targets in therapy to reduce HIV patients’ systemic activation status [89].

#### 3.1.4. Loss of Proteostasis

The deterioration of protein homeostasis (proteostasis) is a prevalent characteristic of ageing and disease, marked by the formation of nonnative protein aggregates in various organs. The proteostasis network consistently mitigates protein aggregation through a variety of macromolecular mechanisms that preserve proteome integrity throughout subcellular compartments and tissues, hence promoting a healthy lifespan [90]. Consequently, eukaryotic cells have evolved various methods to degrade certain proteins, which are integral to the overall protein quality control process. The ubiquitin-proteasome is a system wherein a protein substrate designated for destruction is covalently changed by polyubiquitin chains and subsequently guided to the proteasome for proteolysis. It is not only responsible for the disposal of damaged or misfolded intracellular proteins but also serves as necessary in various cellular pathways, including cell cycle progression and signalling pathways, where the selective degradation of protein components in a time-dependently and spatially regulated manner is needed for maintaining normal cellular functions [91]. The primary purpose of these proteins is to regulate the host’s ubiquitin-dependent proteasomal breakdown system by utilising various routes. HIV-1 proteins modify the particular characteristics of cellular E3 ligases to facilitate viral replication; for instance, the ubiquitin-proteasome system is indispensable for NF-κB signalling, which HIV-1 exploits, as well as for the last stage of assembly and dissemination of viral fragments from the cells that are infected [92].

Vif recruits a SCF-like E3 ubiquitin ligase complex that comprises Cullin5, Rbx2, Elongin B, and Elongin C to degrade APOBEC3 family members, including A3G. Through its N-terminal domain, Vif binds directly to A3G, promoting polyubiquitination and destruction by the 26S proteasome. Vif interacts with CBF-β to stabilise the process, increasing A3G degradation efficiency and inhibiting transcription [93,94]. Vpx likewise targets SAMHD1, a deoxynucleoside triphosphohydrolase that depletes intracellular dNTPs to inhibit HIV replication; Vpx interacts with DCAF1 and SAMHD1 to polyubiquitinate and degrade SAMHD1 via the CUL4A-DDB1-DCAF1 E3 ubiquitin ligase complex. This restores dNTP availability, enhancing reverse transcription in non-dividing myeloid cells and resting CD4+ T cells [95,96]. Vpu opposes BST-2/Tetherin, which anchors newly produced virions to the host cell membrane to prevent virion release. Vpu interacts with BST-2 via transmembrane domains and recruits the SCF-β-TrCP E3 ubiquitin ligase complex. BST-2 is ubiquitinated and endo-lysosomally degraded, enabling viral particle release. Thus, Vpu enhances CD4 receptor degradation through the endoplasmic reticulum-associated degradation pathway, where β-TrCP-mediated ubiquitination drives CD4 towards proteasomal degradation [97,98].

Additionally, it has been found that Tat can inhibit IRF1’s transcriptional activity and evade host immunological responses by inducing its ubiquitination and proteasomal destruction through Hdm2. Tat-induced ubiquitination, through the GluN2A/Akt/Mdm2 pathway, contributes significantly to HIV-associated cognitive disorder (HAND), which is defined by defective N-methyl-D-aspartate receptors (NMDARs) [99,100].

#### 3.1.5. Disabled Macroautophagy

Every cell in the eukaryotic kingdom undergoes autophagy, a process primarily involved in degradation. It maintains cellular homeostasis, adapts to changing nutritional conditions, removes unnecessary and damaged organelles, and recycles cytoplasm to produce macromolecular components and energy when stressed [101]. Furthermore, autophagy is involved in antigen presentation, the clearance of invasive microorganisms, and the prevention of harmful protein accumulation, all of which contribute significantly to cytoprotection. Macroautophagy is the most common type of autophagy; during this process, the cell builds an autophagosome out of a phagophore, a double-membrane sequestering compartment [102].

Substantial evidence indicates that diminished autophagy plays a significant role in the ageing process. Research across various organisms has demonstrated that autophagic activity inherently diminishes with advancing age. Furthermore, cells derived from long-lived species, including humans, typically exhibit heightened baseline rates of autophagy. Moreover, deficiencies that lead to diminished autophagic activity have been associated with the acceleration of the ageing process and the onset of age-related disorders [103]. The decreased activity of macroautophagy was initially regarded as a specific instance of compromised proteostasis. Nevertheless, macroautophagy is not limited to the degradation of proteins; it also encompasses the targeting of complete organelles and non-proteinaceous macromolecules, thereby warranting its consideration as a distinct phenomenon [39].

HIV infection and its viral proteins impair the autophagy mechanism in multiple cell types, such as microglia, macrophages, astrocytes and T cells. The HIV-associated neurocognitive disorder is increasingly prevalent despite ART, potentially due to accelerated brain ageing, enhanced chaperone-mediated autophagy that prematurely degrades vital neuronal machinery, or a combination of both factors [43]. In this direction, Laura Cheney et al. showed that after 24 h of therapy, Nef and ART reduce autophagosomes via distinct pathways. Nef promotes the destruction of autophagosomes without triggering their production, whereas ART obstructs the formation of autophagosomes. The combination of Nef and ART further reduces autophagosomes by causing both defects. Moreover, extracellular Nef and ART impede the lysosomal degradation of p62, suggesting that Nef and ART differentially influence bulk and selective autophagy [104]. While certain research suggests that autophagy is harmful to the virus, others assert that HIV intentionally stimulates this process to enhance its infectivity. Sergio Castro-Gonzalez et al. demonstrated that autophagy impedes HIV replication, markedly decreasing virion generation. However, HIV-1 employs its auxiliary protein, Nef, to mitigate this constraint. They discovered that Nef also inhibits the initiation of autophagy by augmenting the interaction between BECN1 and its antagonist BCL2, a process contingent upon the cellular E3 ligase PRKN. The findings indicated that HIV-1 is vulnerable to autophagy inhibition and identified Nef as the principal inhibitor of this antiviral mechanism [105]. Nef, gp120, and Tat have also demonstrated interactions with molecules that require appropriate autophagy, contributing to HIV-associated cardiomyopathy. Tat inhibits autophagy by decomposing autophagic proteins such as SQSTM1/p62 and LC3-II, obstructing the autophagosome during the growth phase. In contrast, viral gp120 facilitates the expansion and maturation stages of autophagosomes by increasing the levels of ATG7, Beclin 1, LAMP1, and LC3-II. Ultimately, Nef interacts with Beclin-1 and Rab7 to obstruct autophagy during the fusion phase. The interplay of these alterations in autophagy leads to an increased quantity of autophagosomes that are unable to fuse for the degradation of defective proteins [106].

### 3.2. Antagonistic Hallmarks of Ageing

The antagonistic hallmarks exhibit contrasting effects based on their intensity; they provide protection at low levels but become harmful when chronic or intensified, a phenomenon that occurs with ageing. This category will discuss cellular senescence, mitochondrial dysfunction, and deregulated nutrient-sensing [40].

#### 3.2.1. Cellular Senescence

At cellular senescence, cells are permanently unable to divide even when exposed to mitogenic stimuli and ideal growth conditions. Because cell survival pathways, such as the BCL-2 family of antiapoptotic proteins, are upregulated in senescent cells, they are more resistant to apoptotic cell death, even when exposed to exogenous stress [107]. Various mechanisms are activated to promote senescence, and these pathways differ according to the trigger. Some examples of such triggers include DDR, telomere shortening, and oncogenic signalling pathway activation, all of which activate p53. One of the key regulators of p53 function is the E3-ubiquitin ligase MDM2. Because MDM2 directly binds to p53 and targets it for degradation via the ubiquitin-proteasome system, it functions as a negative regulator of p53 activity. By limiting p53 stability, MDM2 prevents p53-mediated transcription of genes involved in cell cycle arrest, senescence, and apoptosis. One more endogenous p53 inhibitor, MDMX (or MDM4), enhances MDM2 activity and suppresses p53 transactivation [108]. Various factors, such as inflammatory cytokines and chemokines that can enter the circulation and promote systemic inflammation, as well as growth factors that primarily act in a paracrine manner to influence the local tissue microenvironment, are released by senescent cells with a senescence-associated secretory phenotype (SASP) [109]. A pair of transcription factors, NF-κB and CCAAT/enhancer binding protein β (C/EBPβ), mainly control the inflammatory SASP. Depending on the situation, p53 can regulate NF-κB activity either positively or negatively, resulting in a complex interaction between the two proteins. It has been demonstrated that p53 seems to limit the stress-inducible kinase, p38-MAPK, which is involved in NF-κB activation during senescence. It was recently demonstrated that ATM and ataxia-telangiectasia-mutated and Rad3-related (ATR), which are DNA damage sensors, activate NF-κB via GATA Binding Protein 4 (GATA4), hence promoting the SASP [110]. By controlling cellular destiny and tissue homeostasis, retinoblastoma protein (pRb) is deemed necessary in senescence programs. Proliferative signals are integrated with cell cycle checkpoints through the pRb-E2F pathway, which ensures correct cellular division and genomic stability [10]. The onset and persistence of senescence are cellular destiny decisions that pRb modulates by controlling E2F activity, thus preventing the transcription of genes required for cell cycle progression [111]. Moreover, pRb promotes senescence stability by recruiting histone methyltransferases to E2F target promoters, enriching repressive H3K9me3 marks and reducing activating H3K4me3 modifications. It also contributes to the formation of senescence-associated heterochromatin foci (SAHFs), facilitating large-scale chromatin compaction and long-term transcriptional silencing [112]. There are two types of cellular senescence: premature and telomere-dependent, often known as replicative senescence, which depends on age [113].

In the context of HIV/AIDS, the concept of “immunosenescence” is of particular interest. Chronic HIV infection is characterised by immune activation. An important component of HIV pathogenesis, immune activation happens even when ART is successful in inpatient management. T cells naturally undergo age-related changes, but activation and inflammation caused by chronic infections like HIV provide an environment where replicative senescence of T cells can occur more quickly. CD4 T cells are infected by HIV and severely reduced in number, leading to immunodeficiency and, ultimately, AIDS [114]. Recently, Lara-Aguilar et al. demonstrated that low-level viremia of 50 and 200 copies/mL reduces cytotoxic activity and T-cell malfunction, affecting cytokine production and inability to regulate and eradicate infected cells. Senescence markers increase, indicating immunological memory loss and a reduction in immune cell proliferation. Accelerated immune ageing may increase comorbidity risk. These data clearly suggest increased HIV patient surveillance to detect possible subsequent issues [115]. In addition, the development of cancerous tumours is closely related to immunosenescence, a process that worsens with age and is characterised by changes in the shape of lymphoid organs and a decrease in the efficiency of immune cells [109]. The most prevalent changes in immunosenescence include decreased activity and antibody generation by B cells, a buildup of memory T cells with diminished diversity and functionality, a decrease in the efficacy of innate immune cells, a reduction in naive T cell production due to thymic involution, including neutrophils and natural killer cells [116].

The disparity of immune cell ratios, especially for T cells, is greatly influenced by thymic involution. One type of thymic tissue is epithelial, whereas another is the non-epithelial perivascular space, which does not undergo thymopoiesis. As the thymus undergoes atrophy, the epithelial compartments progressively diminish, resulting in the gradual occupation of the perivascular space by adipose tissue and fibroblasts, replacing the functional thymic tissue. This process culminates in a reduction of naïve T cells, an elevation of peripheral late-differentiated memory T cells, and a decreased migration of naïve T cells to peripheral tissues [113]. The vicious cycle of infection-associated antigen generation and the loss of control over HIV infection is brought about by persistent HIV infection. It is worth mentioning that inhibitory signals of T cell activation, such as PD-1, TIGIT, and LAG-3, have been associated with exhausted T cells, ongoing HIV infection, and disease progression among those on ART. The release of pro-inflammatory cytokines happens after the thymus has been involuted, which is associated with chronic inflammation and an increased vulnerability to infections, autoimmune illnesses, cardiovascular conditions, and other consequences seen in the elderly. Additionally, immunosenescent HIV-positive patients advance to AIDS more rapidly [48]. In the same direction, Soares et al. found that long-term HIV infection accelerates the immune system’s ageing process. An inactive subpopulation of NK cells, CD3-CD56-CD16+, is produced by chronic HIV infection. The frequency of global DNA methylation of NK cells increases in advanced HIV infection. HIV hinders NK function by causing cytotoxicity, unregulated ROS, and cytokine generation [117].

Even in patients who are responding well to ART, immunosenescence increases the risk of age-related complications, including cardiovascular disease, neurocognitive problems, osteoporosis, fragility, and several cancers. Patients with HIV experience accelerated immune system ageing due to chronic inflammation and immunological activation, which causes immune exhaustion and a decrease in T cell regeneration ability. Immunosenescence also forecasts higher morbidity and death due to deficient CD4+ T cell recovery and worse immunological reconstitution after ART. In addition to AIDS-related problems like atherosclerosis and liver fibrosis, non-AIDS-related comorbidities, including renal impairment and chronic inflammatory state mediated by senescent cells, worsen end-organ damage [118].

#### 3.2.2. Mitochondrial Dysfunction

HIV patients demonstrate increased ROS production in monocytes, along with significantly elevated levels of oxidised nucleic bases, such as 8-oxoguanine (8-oxoG), and lipid-based peroxidation byproducts, including malondialdehyde in plasma and alkanes in breath output [119]. Out of these, 8-oxoG holds particular significance in telomere research. Telomeric repeats exhibit susceptibility to oxidative damage due to their high guanine triplet content, making them more prone to oxidative DNA lesions compared to other genomic regions [120]. Among the bases at this level, guanine exhibits the highest susceptibility to oxidation [121]. Subsequently, ROS directly induces DNA damage through the increased generation of 8-oxoG lesions in telomeres, leading to the oxidation of nucleoside bases [122]. The preferential incorporation of 8-oxodGTP opposite adenine during the replication of the 5–15 kb telomere duplex would result in the transformation of TTAGGG repeats into GTAGGG and TGAGGG repeats. Secondly, telomerase is capable of incorporating 8-oxodGTP during the process of telomere extension. Similar to the majority of DNA polymerases, it exhibits a preference for misincorporating the oxidised dGTP opposite rA [123]. Consequently, through these conversions, oxidative stress can result in DNA damage and promote senescence.

**Figure 3:**
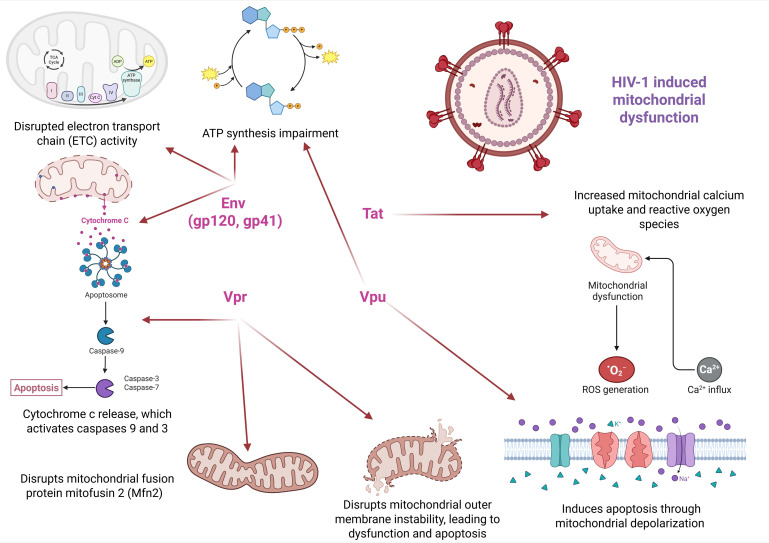
HIV-1-induced mitochondrial dysfunction [128,129] (Created with BioRender.com). Legend: ATP—adenosine triphosphate; Ca^2+^—calcium ion; ETC—electron transport chain; Env—envelope proteins; gp120—glycoprotein 120; gp41—glycoprotein 41; HIV—human immunodeficiency virus; Mfn2—mitofusin 2; O_2_^−^—superoxide anion; ROS—reactive oxygen species; Tat—transactivator of transcription; Vpr—viral protein R; Vpu—viral protein U.

Moreover, total antioxidant capacity, GSH/GSSG ratio in epithelial lung fluid, and blood GSH levels are all negatively impacted in HIV infection [119]. Moreover, one enzyme that defends against oxidative stress, manganese superoxide dismutase, can have its expression significantly downregulated by Tat. Additionally, Tat can trigger OS by activating spermine oxidase, which in turn activates the Nrf2/ARE pathway and causes glutathione exhaustion by stimulating N-methyl-D-aspartate (NMDA) receptors. Activation of NMDA receptors leads to calcium influx and mitochondrial dysfunction, contributing to oxidative stress. HIV-1 Tat enhances intracellular ROS levels via stimulating the production of two key NADPH oxidases, NOX2 and NOX4. The activation of the NF-κB signalling pathway by TNF can increase the generation of ROS by lowering mitochondrial superoxide dismutase, and this activation can be Akt-dependent. The NOX2 expression is associated with this process [124]. Furthermore, Tat-induced suppression of SIRT1 and SIRT3 results in recurrent endoplasmic reticulum stress and mitochondrial malfunction. These cellular events trigger the stimulation of the intrinsic pathway, which causes cell death [125]. Persistent oxidative stress contributes to the alteration of mitochondrial constituents, proteins, lipids, and mitochondrial DNA. Lipid peroxidation directly affects the structural integrity of mitochondrial membranes and causes increased permeability and fluidity [126]. In the same direction, oxidative stress impairs the electron transport chain, which contributes to decreased ATP synthesis and the generation of more oxidative stress [127]. Basically, a persistent oxidative stress loop is formed that amplifies mitochondrial dysfunction and promotes the activation of cellular apoptosis pathways. Figure 3 is an overview of the HIV-1 proteins’ effects on mitochondrial homeostasis.

Moreover, different cell types have variable control of mitochondrial function when HIV is present. For example, mitochondrial membrane potential (ΔΨm) is lower in HIV-positive individuals who have not yet received ART compared to healthy individuals who do not have HIV, and there is a negative relationship between ΔΨm and the proportion of lymphocytes that undergo cell death. In patients infected with HIV who have not yet received ART, there is a positive correlation between the number of CD4+ T cells and changes in ΔΨm. The actions of proteins encoded by viruses are likely contributing to mitochondrial dysregulation [128].

#### 3.2.3. Deregulated Nutrient-Sensing

The discovery that reduced calorie intake or impaired nutrient signalling delays the ageing process suggests a possible relationship between unregulated nutrient-sensing and HIV-1 infection. A major contributor in this context is the insulin-like growth factor (IGF-1), which is primarily produced by the liver in response to growth hormone (GH) secreted by the brain. Age-related reductions in IGF-1 signalling are supported by mutagenesis of proteins involved in IGF-1 signalling, including GH, IGF-1 receptor, AKT, FOXO transcription factors, and mTOR, which have been associated with longevity. The GH release assay demonstrated that Tat inhibited the secretory function of neuroendocrine cells [130].

In particular, mTOR is necessary for the HIV-1 life cycle since it controls metabolic and signalling pathways for virus entrance, replication, and latency. Glycolytic and pentose phosphate pathways, as well as other metabolic pathways, including amino acid absorption, lipid metabolism, and autophagy, are regulated by mTOR and its downstream effectors, which play an important role in regulating host cell metabolism [131]. HIV-1 infection often promotes viral integration and replication by increasing mTORC1 activity in both productively infected and bystander host cell populations. Many model cell lines, including Jurkat, HeLa, and HEK293, as well as peripheral blood mononuclear cells, activate mTORC1 after HIV-1 infection [132]. The persistent activation of the mTOR complex promotes anabolic metabolism and protein synthesis [133]. On the one hand, it supports viral replication. On the other hand, it contributes to metabolic dysregulation in host cells and leads to insulin resistance, lipid accumulation, and impaired autophagy, all of which are hallmarks of metabolic syndrome observed in HIV-infected individuals [134].

Sirtuins modulate lipid and glucose metabolism in reaction to physiological fluctuations in energy levels, thereby serving as necessary mediators of the network governing energy homeostasis, which in turn influences healthspan [135]. The decline in sirtuin activity impairs mitochondrial biogenesis, fatty acid oxidation, and antioxidant defences, leading to dysregulated nutrient-sensing in HIV patients.

Viral Tat suppresses the AMPK signalling pathway via the NAD+/SIRT1 pathway, which, in turn, stimulates the transactivation of the HIV-1 LTR [136]. AMPK is an energy regulator in cells. Upon activation in response to a drop in energy status, it conserves ATP by turning off biosynthetic pathways and increases ATP generation by upregulating the expression or activity of catabolism-related proteins. At the level of the whole body, AMPK also controls the balance of biological energy [137]. Furthermore, miR-217 plays a part in Tat-induced LTR transactivation through decreasing SIRT1 [136].

### 3.3. Integrative Hallmarks of Ageing

The integrative characteristics involve tissue homeostasis and initially comprised the two additional hallmarks of ageing: stem cell depletion and modified intercellular communication. As per the updated classification, this category will discuss stem cell exhaustion, altered intercellular communication, chronic inflammation, and dysbiosis [40].

#### 3.3.1. Stem Cell Exhaustion

Stem cell exhaustion denotes the gradual decline in progenitor cell functionality and regenerative ability over time, resulting in reduced tissue repair and preservation. Stem cell division for tissue replenishment throughout life is hindered by telomere shortening, DNA damage accumulation, and changes in the niche microenvironment, leading to diminished proliferation and altered differentiation potential [138].

In HIV patients, bone marrow niche dysregulation due to direct and indirect HIV impacts induces haematological abnormalities. Haematopoietic stem/progenitor cells (HSPCs) are maintained in the bone marrow niche, which is a complex, multicellular environment. The bone marrow niche cells support HSPCs and regulate their quiescence, self-renewal, and differentiation by biochemical and molecular signals and interactions between cells [139].

In the setting of HIV infection, a decline in stem-cell functioning is apparent across many stem-cell regenerative capacities. The depletion of haematopoietic progenitors (CD34) and naive T cells is seen, leading to consequences potentially associated with systemic immune activation and inflammaging [43]. In fact, HIV infection is a pathology marked by increased levels of systemic and persistent inflammation (as detailed in Section 3.3.3). Experimental studies in mice indicated that chronic inflammation or repeated acute inflammatory episodes may prematurely deplete the regenerative capacity of the haematopoietic system and impair its functionality [140]. Haematopoiesis effectively manages isolated acute stress events without a notable decrease in long-term haematopoietic stem cells (HSC), while chronic or recurrent acute inflammation may result in HSC attrition [141].

#### 3.3.2. Altered Intercellular Communication

During various stages of the viral life cycle, HIV-1 viral proteins may trigger several signalling networks throughout the target cell. These signaling changes not only influence the infected cell’s fate but also disrupt communication between immune and non-immune cells, contributing to systemic inflammation and immune dysregulation, features of altered intercellular communication in aging. There are many different viral proteins, and they all contribute to the pathogenesis and persistence of HIV-1 [142]. The HIV-1 life cycle requires viral proteins that modulate cell signalling and serve as molecular switches, allowing the virus to rely on both host cell machinery and viral machinery for its replication and pathogenicity [143]. However, for the purpose of this review, we are going to discuss the most important recent findings.

The NF-κB signalling is activated by HIV-1 proteins during the initial phases of infection. In order to activate the NF-κB and AP-1 signalling pathways and boost the HIV-1 LTR promoter, HIV-1 Vpr increases the phosphorylation and polyubiquitination of transforming growth factor-β-activated kinase 1 (TAK1) [144]. The HIV-1 Tat transactivator promotes NF-κB activation by interfering with its inhibitor, IκB-α. Under normal conditions, IκB-α binds to the NF-κB complex and prevents its nuclear translocation. Tat disrupts this interaction, triggering the release and nuclear entry of NF-κB, which subsequently initiates the transcription of target genes. In addition, Tat enhances the transcriptional activity and DNA-binding affinity of the p65 subunit of NF-B by associating with it. For IκB-α and p65 interaction, respectively, and for maintaining NF-κB activity, the arginine- and cysteine-rich regions of Tat are necessary. Tat binds to the NF-κB promoter region of the macrophage inflammatory protein-1α (MIP-1α) gene, facilitating the recruitment of the p65 subunit of NF-κB and triggering the displacement of its inhibitor. Thus, MIP-1α gene expression is enhanced through a p65-dependent mechanism, along with the upregulation of other NF-κB-responsive genes. [145]. Chronic activation of NF-κB is observed in monocytes, macrophages, and microglia infected with HIV-1, resulting in increased expression of NF-κB-responsive genes, such as pro-inflammatory cytokines, adhesion molecules that bind to cells, and chemokines [145]. By blocking the degradation of IκBα, several viral proteins inhibit NF-κB activity. For instance, in order to sustain infection over an extended period of time, HIV-1 alters the activity of IκBα. By attaching to the E3 ligase β-transducin repeat-containing protein (βTRCP) within the E3 ubiquitin ligase complex, which contributes to the controlled degradation of IκBα, Vpu prevents the proteasome-dependent degradation of IκBα. So, HIV-1 causes infected T cells to undergo apoptosis by lowering the levels of cellular anti-apoptotic proteins, including BCL-XL and TRAF1, which are dependent on NF-κB [146]. Activation of the NF-κB pathway by HIV proteins leads to increased secretion of pro-inflammatory cytokines and chemokines, which act on neighboring immune cells to drive chronic inflammation and immune system hyperactivation [147].

In the promonocytic cell line U937, tumour necrosis factor receptor-associated factor (TRAF) was shown to activate the HIV-1 LTR even in the presence of mutations in NF-κB binding sites. This suggests the existence of an alternative, NF-κB-independent pathway by which TRAF can promote HIV-1 transcriptional activation. P38 MAPK is activated by inputs downstream of TRAF2 and TRAF5, according to research by Ryouichi Horie et al., and this activation directly phosphorylates C/EBPβ. Furthermore, the activation of p38 MAPK strongly promotes the amplification of HIV-1 gene expression that is mediated by C/EBPβ [148]. Nef also seems to rely on p38/MAPK activation to control apoptosis through upregulating the programmed death-1 surface protein [149]. Nef increases apoptosis by decreasing the expression of Bcl-2 and Bcl-XL, two proteins that prevent cell death [150].

HIV-1 infection often promotes viral integration and replication by increasing mTORC1 activity in both productively infected and bystander host cells. Also, viral proteins (such as Gag) need mTORC1 activity to be synthesised optimally [132]. In order to increase cell survival, Env binds to CD4 cell surface receptors, which activate Akt and suppress p38 MAP kinase activation. Additionally, it induces cell death by interacting with CCR5 receptors, which promotes the production of programmed cell death protein 1 (PD-1), Fas, and FasL. Reducing the p38 expression of caspases, Akt activation mitigates the subsequent increase in apoptosis [151]. Interestingly, there have been reports that Nef can suppress cell death by phosphorylating Bad, thereby inhibiting its proapoptotic activity. It also induces the direct activation of PAK and PI3Ks [152]. Moreover, Hyung Joon Cho et al. demonstrated that HIV alters intercellular communication by inducing PD-L1 expression on antigen-presenting cells (APCs), primarily through activation of the PI3K/Akt intracellular signaling pathway. The upregulated PD-L1 then interacts with PD-1 on HIV-specific CD8+ T cells, leading to their functional exhaustion—marked by reduced proliferation and cytokine secretion—thereby impairing effective antiviral immune responses [153].

There have been reports of many HIV-1 proteins interacting with p53, each with a distinct set of effects. By attaching to the N-terminal region of p53 (transactivation domain), Nef either decreases p53 expression or reduces its function. This prevents p53 from binding to DNA and from having the ability to activate transcription, which in turn inhibits its apoptotic function. Moreover, Env controls cell death by inducing p53 phosphorylation in HIV-infected primary macrophages, whereas the Vif protein has been reported to stabilise and activate p53, contributing to cell cycle dysregulation [154]. Vif maintains p53 in a stable and active state. Infected cells are able to sustain HIV-1 replication through inducing G2 phase arrest. In neurones, Tat was found to upregulate miR-34a [155]. Emerging evidence suggests that HIV-mediated regulation of p53 and apoptosis may vary depending on the stage of infection and treatment status. Nef, Tat, and Vpr are viral proteins that can inhibit apoptosis in the early phases of the viral life cycle in order to increase cell survival and facilitate viral reproduction [150,156,157]. In contrast, in chronic infection, particularly in the absence of ART, viral proteins tend to activate p53 and induce apoptosis, contributing to immune cell depletion and systemic inflammation [158]. These differences reflect a dynamic viral strategy: initially favoring cell survival to maximise viral replication, and later promoting cell death and immune dysregulation, contributing to disease progression. However, to date, there are no reports on the impact of ART on p53 function. This would be an interesting research direction, particularly important in the context of the development of new therapeutic strategies.

**Figure 4:**
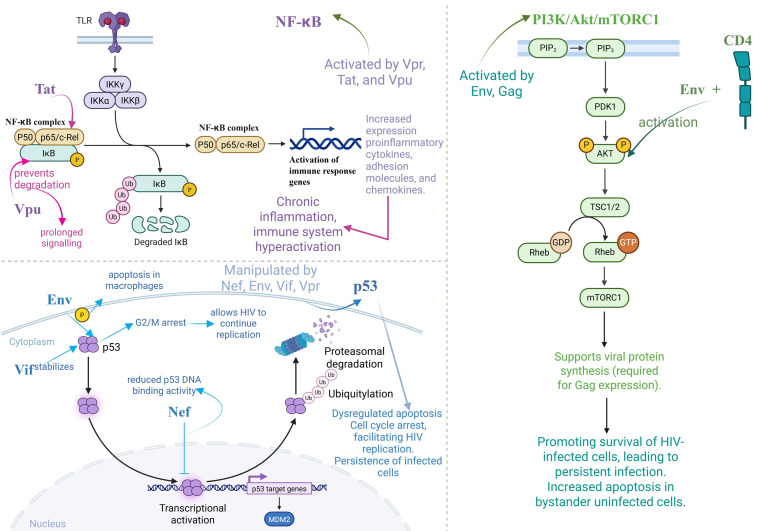
NF-κB, PI3K/Akt/mTORC1, and p53 signalling pathways in HIV-1 infection (Created with BioRender.com). Legend: AKT—protein kinase B; CD4—cluster of differentiation 4; Env—envelope proteins; Gag—group-specific antigen; GDP—guanosine diphosphate; GTP—guanosine triphosphate; HIV—human immunodeficiency virus; IKB—inhibitor of nuclear factor kappa-B; IKKα—IκB kinase alpha; IKKβ—IκB kinase beta; IKKγ—IκB kinase gamma; mTORC1—mechanistic target of rapamycin complex 1; MDM2—mouse double minute 2 homolog; Nef—negative factor; NF-κB—nuclear factor kappa-light-chain-enhancer of activated B cells; PDK1—3-phosphoinositide-dependent kinase 1; PI3K—phosphoinositide 3-kinase; PIP_2_—phosphatidylinositol 4,5-bisphosphate; PIP_3_—phosphatidylinositol (3,4,5)-trisphosphate; p53—tumor suppressor protein 53; Rheb—Ras homolog enriched in brain; TSC1/2—tuberous sclerosis complex 1/2; Tat—transactivator of transcription; Ub—ubiquitin; Vif—virion infectivity factor; Vpr—viral protein R; Vpu—viral protein U.

The altered communication in HIV-1 infection concerning the NF-KB, PI3K/Akt/mTORC1, and p53 signalling pathways is summarised in Figure 4.

Moreover, the Janus kinase/signal transduction and transcription activation of transcription (JAK/STAT) pathways are involved in every step of the immune response, from defending against infections to preserving immune tolerance, from enhancing barrier function to preventing cancer. Also, extracellular mechanistic signalling is reliant on the JAK/STAT pathways, which may mediate mechanistic signals that impact the immune system and the course of disease [159]. HIV persistence, which is associated with viral DNA assembly in vivo and T cells, is mediated by the JAK/STAT genes. In HIV-positive individuals, the levels of CCR5 are decreased by the JAK inhibitors tofacitinib and ruxolitinib. Consequently, HIV infection, integration, and immunological response are all linked to the JAK/STAT signalling system [160]. According to Siobhan Gargan et al., the Vif blocks the efficient signalling of IFN-α by destroying important elements of the JAK/STAT pathway. HIV-1 IIIB strain induced STAT1 and STAT3 breakdown in a Vif-dependent manner, as demonstrated by the lowered levels of these proteins in HEK293T cells expressing Vif. A functional result of this HIV-1-mediated immune bypass approach was shown when myeloid ThP-1 cells were infected with HIV-1 IIIB, which decreased the activation of the anti-viral gene ISG15 through IFN-α but had no effect on MxA [161]. In both Tat-expressing and HIV-infected astrocytes, there was an upregulation of STAT3 expression and phosphorylation. STAT3 and its phosphorylation induced a favourable reaction in the transcription and protein production of GFAP, Egr-1, and p300 [162]. Manipulation of the JAK/STAT pathway by HIV proteins impairs interferon signaling and antiviral gene expression, weakening the antiviral state of neighboring cells and facilitating viral persistence and immune evasion [163].

Moreover, Hyung Joon Cho et al. proposed that injury signals are transmitted from infected pericytes to adjacent cells through gap junction-mediated intercellular communication. In the context of various gap junction proteins studied, HIV infection in human brain pericytes specifically elevated the expression of connexin 43. Additionally, HIV infection improved functional gap junction-mediated intercellular communication in pericytes, a process whose significance was demonstrated in experiments where the inhibition of gap junctions by carbenoxolone reduced HIV infection. Furthermore, an extracellular ATP release assay indicated that HIV may contribute to the opening of connexin-containing hemichannels [153].

#### 3.3.3. Chronic Inflammation

Initially, cellular damage is counteracted by the induction of cell cycle arrest in ageing cells. Senescent cells then develop a senescence-associated secretory phenotype (SASP), characterised by the release of pro-inflammatory molecules such as IL-6, IL-8, matrix metalloproteinases, monocyte chemotactic protein, and insulin-like growth factor binding proteins. These factors serve as signals that recruit immune cells to eliminate the damaged cells. However, due to immune dysfunction, this clearance process becomes inefficient. As a result, senescent cells accumulate and contribute to a chronic inflammatory state [164]. The NF-κB pathway, a central regulator of inflammation, is further activated by both inflammation and oxidative stress associated with ageing. This persistent activation of SASP factors, along with self-antigens and accumulated cellular debris, drives a state of chronic, low-grade inflammation known as “inflammaging”. Over time, this response can disrupt cellular and tissue homeostasis and become self-destructive [164,165].

The immune system’s persistent activation in patients with HIV results in chronic inflammation, which is linked to a variety of comorbidities, such as cardiovascular disease, liver dysfunction, and a predisposition to cancer. This is the case even in the presence of ART [53,166]. The molecular mechanisms involved include activation of the NLRP3 inflammasome, which triggers the secretion of IL-1β and IL-18, promoting systemic inflammation and intestinal barrier dysfunction [166]. Additionally, systemic inflammation is also significantly influenced by microbial translocation, which is a result of intestinal barrier dysfunction. Microbial translocation refers to the process by which bacteria or bacterial products, normally confined to the intestinal lumen, cross the epithelial barrier into the bloodstream, ultimately promoting inflammation and disease pathogenesis [167]. This translocation activates TLR4 and enhances the stimulation of monocytes and macrophages [168,169]. Therefore, therapeutic interventions that focus on the regulation of these inflammatory pathways, such as the inhibition of the NLRP3 inflammasome, the blockade of checkpoint proteins, and the restoration of gut microbiota balance, have the potential to be effective strategies for reducing chronic inflammation in HIV patients [166,170].

Immune checkpoint proteins, including PD-1, TIM-3, and BTLA, also contribute to the maintenance of an inflammatory status by promoting cellular exhaustion and inhibiting T lymphocyte function [171,172].

Another important component is the activation of proinflammatory macrophages, which secrete cytokines such as TNF-α, IL-6, and IL-8, thus intensifying oxidative stress and tissue damage [173]. Furthermore, HIV viral proteins, including gp120, Tat, and Nef, may contribute to inflammation by activating NF-κB-dependent proinflammatory pathways and stimulating the production of ROS [53]. The HIV-1 LTR is modulated by both viral proteins and host factors, including NF-κB, which is active during viral infection. The HIV-1 genome possesses two repeating tandems in the LTR region, serving as binding sites for NF-κB and ranking among the most conserved sequences within the HIV-1 genome. The cytoplasmic domain of HIV-1 Env has recently been shown to trigger the NF-κB pathway. In contrast, the HIV-1 protein Vpu has been found to suppress viral replication via regulating the NF-κB signalling pathway and consequent immunological responses [99].

The inflammatory process decreases the antioxidant capacity of cells and causes oxidative stress. Excessive free radicals interact with cell membrane fatty acids and proteins, resulting in permanent functional impairment [174]. The accumulation of ROS can lead to DNA damage and the activation of DDR pathways, resulting in various cellular responses, including senescence [175]. The induction of stress-induced premature senescence is initially marked by random DNA damage throughout the genome, followed by the activation of a DNA damage response (DDR). The development of the senescent phenotype is characterised by the repair of most DNA damage within 24 h; however, damage in telomeric regions persists for months, leading to a sustained and unresolved DNA damage response. It is interesting to note that these telomeric lesions happen regardless of telomere length and whether the telomerase enzyme is present or not [176].

#### 3.3.4. Dysbiosis

HIV infection causes negative changes in the gut microbiota, resulting in a dysmicrobial status characterised by a reduction in bacterial diversity, an increase in opportunistic bacteria, and a decrease in beneficial species, such as those that produce butyrate, a metabolite with an anti-inflammatory role [177,178]. Disruption of gut barrier integrity increases intestinal permeability, which further facilitates the translocation of microbial products into the circulation. This, in turn, activates the innate immune system and elevates circulating levels of lipopolysaccharides, IL-6, TNF-α, and other pro-inflammatory cytokines [179,180]. In addition to opportunistic bacteria, fungi such as *Candida albicans* can amplify microbial imbalances, intensifying inflammation and gut barrier dysfunction [181]. ART is essential for the regulation of HIV replication; however, its effects on the intestinal microbiota are convoluted and not always advantageous [180]. Research indicates that the microbiome profile of HIV-positive patients is still distinct from that of healthy individuals despite the fact that ART may partially restore microbial diversity and reduce bacterial translocation [177,178]. Some classes of antiretrovirals, such as non-nucleoside reverse transcriptase inhibitors (NNRTIs) and protease inhibitors (PIs), have been associated with specific changes in the microbiome, including reduction of beneficial species and favouring of some proinflammatory bacteria (*Lachnospira*, *Oribacterium*) [180]. Moreover, some antiretrovirals, such as zidovudine and efavirenz, have been shown to have direct antimicrobial effects on some gut commensal species, which may contribute to the persistence of dysbiosis and activation of the immune system [180]. Another important aspect is that ART fails to fully restore homeostasis of the microbiome, which may have implications for the long-term immune response [179]. Differences in the immune response to ART are correlated with certain microbial changes: immunological non-responders show a more dysbiotic microbiome, characterised by an increase in proinflammatory species such as *Fusobacterium* and *Ruminococcus gnavus* and a decrease in beneficial species such as *Faecalibacterium* and *Bifidobacterium* [179]. This persistence of dysbiosis may partly explain the chronic inflammation and increased risk of comorbidities, including cardiovascular disease and neuroinflammation, in treated HIV patients [182].

Adjuvant interventions, including probiotics, prebiotics, faecal microbiota transplantation, and dietary modifications, are currently being investigated as potential strategies for restoring microbial homeostasis and reducing chronic inflammation in this context. Recent research indicates that these methods may be beneficial in regulating the immune response and enhancing the prognosis of HIV patients, particularly those who fail to achieve a sufficient rate of immunologic recovery with ART [182].

## 4. The Impact of ART on Telomere Biology

Advancements in ART have allowed HIV patients to achieve increased longevity and improved health compared to those diagnosed during the initial phases of the HIV pandemic. The average lifespan and disease-free years for individuals with HIV differ from those without HIV, influenced by factors such as the start of treatment and conventional risk factors [43]. Although several therapeutic options demonstrate similar efficacy levels, variations exist in dosage frequency, tablet quantity, drug interactions, and potential side effects [23]. The evaluation of ART’s impact on telomeres provides a novel, molecular-level insight for enhancing health outcomes in HIV-infected individuals. Telomeres serve as an invaluable biomarker for genomic stability and cellular senescence, indicating the buildup of oxidative stress, chronic inflammation, genomic instability, mitochondrial dysfunction, and exposure to various xenobiotics throughout an individual’s life—factors often exacerbated by HIV infection. Appreciating these molecular interactions could guide the optimisation of therapeutic strategies, mitigate accelerated ageing, and decrease the risk of associated comorbidities in individuals living with HIV [121]. Therefore, in Table 1, we have summarised the most important empirical evidence in this direction regarding the NRTIs.

Both in vitro and in vivo studies have demonstrated that NRTIs, but not NNRTIs, decrease telomerase activity [187,188,189]. Tenofovir demonstrated the highest potency in inhibiting telomerase activity and resulted in the most significant telomere length shortening in vitro at therapeutic concentrations. Peripheral blood mononuclear cells from HIV-infected patients undergoing NRTI-based cART exhibited markedly reduced telomerase activity compared to both HIV-uninfected individuals and HIV-infected patients on non-NRTI-containing cART [190]. Additionally, NRTIs are integrated into telomeric DNA, which can cause chain termination and cell death [188]. Interestingly, Bukic et al. demonstrated that only cART incorporating NNRTI had a statistically significant impact on telomere length; specifically, patients treated with efavirenz exhibited significantly shorter telomeres compared to those receiving nevirapine [191].

Considering that HIV infection induces molecular changes characteristic of accelerated ageing, an additional ART-induced shortening of telomere length could have a detrimental effect on premature immunosenescence, thus increasing the risk of developing associated comorbidities.

Alongside the acceleration of premature ageing due to HIV, ART treatment may also play a role in mitochondrial dysfunction. Early initiation of ART and strict adherence to it have been demonstrated to lower the risk of comorbidities in HIV patients [192]. However, immune phenotyping indicates that long-term ART-treated individuals may still exhibit atypical cellular processes, homeostasis, and cell activation status. This suggests that some ART drugs could potentially exacerbate the HIV-related acceleration of premature ageing [128]. Moreover, the accumulation of mtDNA mutations leads to increased mitochondrial oxidative stress and a reduction in the average lifespan of mitochondria, primarily induced by the class of NRTIs and protease inhibitors (PIs) used to enhance intracellular concentrations of NRTIs [128]. Untreated HIV infection correlates with elevated levels of inflammation, indicated by the presence of inflammatory cytokines, including IL-1β, IL-6, and TNFα. The majority, if not all, of these inflammatory markers, decrease with cART, suggesting that ongoing HIV replication is directly or indirectly accountable for this inflammatory response. The level of inflammation remains elevated even with durable and potentially complete suppression of HIV replication through ART. Several factors likely contribute to the ongoing inflammation during therapy. These include low-level HIV replication in anatomical reservoirs, elevated co-pathogen load, decreased T regulatory and other immunoregulatory cells, translocation of lipopolysaccharides through compromised gut mucosa, and irreversible fibrosis of the thymus and lymphoid tissues [193]. HIV reservoirs are defined as cell types or anatomical sites where replication-competent forms of the virus persist in spite of ART, posing significant challenges to HIV eradication. One possible explanation for residual HIV replication in lymphatic tissues, despite suppressive ART, is thought to be the suboptimal drug penetration into these anatomical sites compared to peripheral blood [194].

Furthermore, HIV-1 infection impairs oxidative phosphorylation, thereby enhancing glycolysis and fatty acid synthesis to support viral replication. ART may worsen metabolic dysregulation even when viral replication is managed, affecting mtDNA synthesis and increasing the production of ROS. The collective effects lead to substantial alterations in oxidative phosphorylation, impacting the metabolism and function of immune cells [129]. Table 2 summarises empirical studies regarding ART drugs and cART.

A first observation is that PI, particularly darunavir-based regimens, may not affect telomere length as markedly as NRTIs; in some cases, even an increase in telomere length was observed [201]. Contrasting is the impact of PI administered to HIV-positive mothers, with newborns showing decreased telomere lengths [202], indicating a potential negative impact of PIs during pregnancy. In summary, the evidence for the PI class is relatively conflicting; there are no studies for all representatives, and long-term clinical studies are needed to accurately assess the impact on telomeres. Future studies should evaluate PIs’ effects across various populations and stratify the results by specific PI drugs to clarify whether certain PIs contribute more to TL changes than others. In the case of INSTI, better maintenance of telomere length can be observed. The evidence in the literature suggests that this class could be a prospective therapeutic option for personalised therapeutic regimens in patients with increased markers of accelerated ageing caused by HIV [197]. However, the effect of INSTIs on TL varies, with some studies showing stabilisation but no significant impact compared to other ART classes [191]. Further research is needed to determine whether integrase strand transfer inhibitor (INSTI)-based ART provides long-term protection for telomere length and aging-related conditions.

Clinically, the most compelling evidence to date suggests that early initiation of ART and sustained virologic suppression may help preserve telomere length and prevent premature shortening [195,196]. In some studies, the rate of telomere shortening stabilised several years after initiation of therapy, but cART does not reverse the damaging effects of HIV. cART only partially reverses HIV-mediated immune defects in infected adults, as demonstrated by the majority of the previously published studies on the role of HIV in the progressive decline of immune system function, which contributes to premature ageing [195,201]. TL may serve as a valuable biomarker for immune recovery and ageing-related outcomes in ART-treated individuals.

However, in HIV-positive children, therapy induces an early, sustained boost in naïve CD4+ T cells, probably indicating higher thymic activity. The underlying causes of these disparities between adults and children remain uncertain. Although the significantly higher thymic export of naïve T-cells in children than in adults may be a contributing factor, additional substantial immune variations between individuals (potentially induced by age, sex, antigenic exposure, and environmental factors) may also play a role [203].

However, the proper assessment of the impact of ART regimens on ageing-related biomarkers individually becomes increasingly challenging due to the administration of cART. The development of enhanced therapeutics and a higher quality of life for HIV patients may be facilitated by an understanding of the distinctive processes that result in ageing biomarkers when HIV infection is combined with ART [128].

## 5. Conclusions

Ageing is a multidimensional biological process marked by a gradual deterioration of physiological integrity, resulting in heightened susceptibility to chronic diseases. Telomeres, repeating nucleotide sequences situated at chromosome termini, preserve genomic integrity and modulate cellular senescence. Progressive telomere attrition correlates with an increased susceptibility to age-related diseases. Chronic infections, such as HIV-1 infection, can accelerate the ageing process via sustained immune activation, chronic inflammation, ROS generation, and direct molecular alterations mediated by viral proteins. The 12 hallmarks of ageing offer a holistic framework for understanding the multiple pathways that contribute to HIV-induced accelerated ageing. These mechanisms are not independent; rather, they are intertwined, with telomeres serving as the most important pillar for cumulative cellular stress and molecular damage. We assert that HIV infection accelerates the ageing process primarily by shortening telomeres, resulting in heightened vulnerability to age-related comorbidities. Comprehending the molecular alterations generated by HIV in the context of ageing hallmarks represents a holistic strategy that may provide novel therapeutic insights to enhance the prognosis of HIV/AIDS patients. ART has converted HIV from a high-mortality illness into a chronic, manageable condition. The influence of ART on telomere dynamics is complex and less understood. ART inhibits viral load, diminishes immunological activation, and may decelerate telomere attrition. Certain therapy regimens, specifically NRTI-based, may produce supplementary cellular stress, including mitochondrial malfunction and oxidative stress, hence facilitating cell senescence and apoptosis. Due to the indispensable function of telomeres in ageing and their susceptibility to viral and therapeutic factors, telomere length is becoming a promising biomarker for evaluating disease progression, treatment effectiveness, and long-term prognosis in individuals infected with HIV. Deciphering the molecular mechanisms governing the interaction between HIV and ageing hallmarks may guide the development of personalised therapeutic strategies designed to mitigate the molecular processes that compromise cellular integrity and expedite ageing. This strategy may enhance therapeutic efforts to regulate viral replication and mitigate the detrimental effects of infection on cellular homeostasis. Consequently, the customisation of treatment may enhance the quality of life for HIV patients, especially considering the abnormal rate of telomere shortening resulting from virus-induced molecular alterations. Incorporating this understanding into clinical practice could transform HIV care by simultaneously targeting infection and dysfunctions related to accelerated ageing.

## Figures and Tables

**Table 1:** Empirical evidence regarding the impact of NRTIs on telomere length.

Drug (Class)	Study Groups	TL Method	Conclusions	Ref.
tenofovir	200 HIV adults: 103 on tenofovir and 97 never exposed to tenofovir	qPCR	No evidence of a significant association between tenofovir exposure and TL when adjusted for confounding variables like age, parental age, race, and duration of HIV infection.	[183]
AZT	179 infants: 94 AZT-exposed; 85 ART-unexposed	qPCR	Infants exposed to AZT during pregnancy had longer telomeres than those unexposed to ART. This may suggest a beneficial effect of maternal ART on neonatal telomere preservation.	[184]
TDF, abacavir, lamivudine	172 HIV+ adults: 67 TDF, 105 non-TDF (69 abacavir, 25 N(t)RTI–sparing regimen, 5 lamivudine	qPCR	Mean TL rose considerably after 2 years. The TDF group gained TL much less than the non-TDF group. TDF exposure had no independent negative impact. Non-TDF patients receiving 2 nucleosides demonstrated considerably less TL improvement compared with N(t)RTI-sparing group or lamivudine.	[185]
tenofovir	128 long-term aviraemic HIV adults: 79 on tenofovir; 49 tenofovir-sparing regimens	qPCR	The inhibitory effect on telomerase by tenofovir in long-term aviraemic HIV adults may account for TL shortening observed in CD8+ T cells. The absence of telomere shortening in the CD4+ compartment, along with the reduction in telomerase activity, can be attributed to both the inhibitory effects of tenofovir and the diminished proportion of recent thymic emigrant CD4+ cells and PD1 marker expression	[186]

Legend: ART—antiretroviral therapy; AZT—zidovudine; CD4+—cluster of differentiation 4 positive cells; CD8+—cluster of differentiation 8 positive cells; HIV—human immunodeficiency virus; NRTI—nucleoside reverse transcriptase inhibitor; N(t)RTI—nucleotide/nucleoside reverse transcriptase inhibitor; PD1—programmed cell death protein 1; qPCR—quantitative polymerase chain reaction; TDF—tenofovir disoproxil fumarate; TL—telomere length.

**Table 2:** Empirical studies regarding ART drugs and cART on telomere length.

Study Groups	TL Method	Conclusions	Ref.
107 HIV+ patients: before and during suppressive ART	qPCR	TL dropped drastically pre-ART. ART stabilised TL without alteration. Early ART initiation may prevent HIV-associated accelerated ageing and preserve telomere integrity.	[195]
375 children: 94 HIV+; 177 HIV-1-exposed uninfected (HEU) exposed to ART; and 104 controls.	qPCR	HIV+, HEU, and HIV− children had similar LTL attrition rates. Linear regression models found no correlation between children’s LTL and perinatal ART exposure or HIV status. The link between a detectable HIV viral load and a shorter LTL shows that uncontrolled HIV viremia, not ART duration, may accelerate blood telomere attrition.	[196]
325 HIV+ patients, stable cART for >1 year: 207 on PIs, 36 on NNRTIs, and 82 on INSTIs; 147 controls.	qPCR	For HIV+ individuals, the rate of decrease in TL with age was more rapid than that of controls. The likelihood of ageing-related neurological disorders in HIV+ may be reduced by the administration of INSTI-based cART and improved viral control, which may delay TL shortening.	[197]
120 HIV+ on stable triple-drug ART: 60 switched to DTG (INSTI) + 3TC (NRTI); 60 continued on TT with 2 NRTIs + an anchor drug.	MMqPCR	A more significant increase in TL over time was associated with a lower basal BTL. The research indicates that the BTL gain is more significant when the simplification is to DTG + 3TC than when the TT is maintained. This could suggest that DTG + 3TC is a potential strategy for mitigating telomere shortening in HIV-treated individuals, as it may reduce telomerase inhibition.	[198]
90 patients with advanced HCV cirrhosis: HIV/HCV group- 60 patients on stable cART + HCV group- 30 patients	MMqPCR	At baseline, patients who were HIV/HCV coinfected exhibited substantially shorter TL than those who were HCV monoinfected. In HIV/HCV-coinfected patients, there was a substantial increase in TL following the eradication of HCV with DAAs. A modest, non-significant increase in TL was observed in HCV-monoinfected patients. Compensated HIV/HCV-coinfected patients exhibited a substantial increase in TL following HCV treatment.	[199]
176 male HIV patients on cART: 53 patients on INSTI-based, 60 patients on PI-based, 63 patients on NNRTI-based, divided into 53 efavirenz and 10 nevirapine.	qPCR	INSTIs, PIs, and NNRTIs did not significantly affect TL in cART groups. Efavirenz (NNRTI) had shorter telomeres than nevirapine (NNRTI), showing that NNRTIs affect cellular ageing indicators differently. Avoiding efavirenz in cART may reduce HIV-related telomere shortening.	[191]
198 HIV-exposed uninfected children (CHEU) on prophylaxis for one year: 0–7 days nevirapine + day 7-week 50–75 LPV/r + 92 3TC	qPCR	TL shortening occurred in 44.3% of CHEU at week 50, regardless of postnatal prophylaxis drugs. Besides motor skills, TL shortening did not affect growth or neuropsychological function at six years. In week 50 and year 6, the LPV/r and 3TC groups experienced similar telomere shortening.	[200]
31 patients: 15 on DRV/r + RAL (PI+INSTI) and 16 on DRV/r + TDF/FTC (PI+NRTI)	qPCR	After 96 weeks of ART, TL rose considerably, more in DRV/r + TDF/FTC than in RAL, but not statistically significant. This TL increase was connected with CD4+ T-cell recovery and inversely associated with effector memory T-cells, suggesting immunological reconstitution changes T-cells with fewer differentiated phenotypes with longer telomeres. Reduced immune activation is linked to TL recovery, as central memory CD8+ T-cells with CD38+ molecules had a negative correlation with TL growth.	[201]
105 pregnant women: 64 HIV+ on cART and 41 HIV-: 39 PI/r; 47 AZT + 3TC, combined with 27 LPV/r or 19 NFV	qPCR	HIV+ mothers who stopped cART post-partum had lower TL. Like both groups, LTL rose during gestation, but it was more evident in women under 35. TL was independently shorter in HIV+ women using ritonavir-boosted protease inhibitors.	[202]

Legend: ART—antiretroviral therapy; AZT—zidovudine; BTL—blood telomere length; cART—combination antiretroviral therapy; CD4+—cluster of differentiation 4 positive cells; CD8+—cluster of differentiation 8 positive cells; CHEU—HIV-exposed uninfected children; DAAs—direct-acting antivirals; DRV/r—darunavir/ritonavir; DTG—dolutegravir; FTC—emtricitabine; HEU—HIV-1-exposed uninfected; HCV—hepatitis C virus; HIV—human immunodeficiency virus; INSTI—integrase strand transfer inhibitor; LTL—leukocyte telomere length; LPV/r—lopinavir/ritonavir; MMqPCR—monochrome multiplex quantitative polymerase chain reaction; NFV—nelfinavir; NNRTI—non-nucleoside reverse transcriptase inhibitor; NRTI—nucleoside reverse transcriptase inhibitor; N(t)RTI—nucleotide/nucleoside reverse transcriptase inhibitor; PI—protease inhibitor; PI/r—ritonavir-boosted protease inhibitor; qPCR—quantitative polymerase chain reaction; RAL—raltegravir; TDF—tenofovir disoproxil fumarate; TL—telomere length; TT—triple therapy.

## References

[1] Li Y., Tian X., Luo J., Bao T., Wang S., Wu X. (2024). Molecular Mechanisms of Aging and Anti-Aging Strategies. Cell Commun. Signal..

[2] Ariestanti D., Elya B., Hartrianti P., Arifin V., Hana C., Lovina P., Fadhila R. (2024). The Anti-Ageing Potential of Litsea Oppositifolia Stem Extract: Evidence from in Vitro and Ex Vivo Study on Skin Cell Lines. Farmacia.

[3] Oliveros A., Poleschuk M., Cole P.D., Boison D., Jang M.-H. (2023). Chemobrain: An Accelerated Aging Process Linking Adenosine A2A Receptor Signaling in Cancer Survivors. Int. Rev. Neurobiol..

[4] Falzone L., Candido S., Docea A.O., Calina D. (2023). Editorial: Inflammation and Aging in Chronic and Degenerative Diseases: Current and Future Therapeutic Strategies. Front. Pharmacol..

[5] Hamczyk M.R., Nevado R.M., Barettino A., Fuster V., Andrés V. (2020). Biological Versus Chronological Aging. J. Am. Coll. Cardiol..

[6] Statsenko Y., Kuznetsov N.V., Morozova D., Liaonchyk K., Simiyu G.L., Smetanina D., Kashapov A., Meribout S., Gorkom K.N.-V., Hamoudi R. (2023). Reappraisal of the Concept of Accelerated Aging in Neurodegeneration and Beyond. Cells.

[7] Kalmykova A. (2023). Telomere Checkpoint in Development and Aging. Int. J. Mol. Sci..

[8] Frydrychová R.Č., Konopová B., Peska V., Brejcha M., Sábová M. (2024). Telomeres and Telomerase: Active but Complex Players in Life-History Decisions. Biogerontology.

[9] Monsen R.C., Chakravarthy S., Dean W.L., Chaires J.B., Trent J.O. (2021). The Solution Structures of Higher-Order Human Telomere G-Quadruplex Multimers. Nucleic Acids Res..

[10] Wu S., Jiang L., Lei L., Fu C., Huang J., Hu Y., Dong Y., Chen J., Zeng Q. (2023). Crosstalk between G-Quadruplex and ROS. Cell Death Dis..

[11] Rossiello F., Jurk D., Passos J.F., d’Adda di Fagagna F. (2022). Telomere Dysfunction in Ageing and Age-Related Diseases. Nat. Cell Biol..

[12] Guo J., Huang X., Dou L., Yan M., Shen T., Tang W., Li J. (2022). Aging and Aging-Related Diseases: From Molecular Mechanisms to Interventions and Treatments. Signal Transduct. Target. Ther..

[13] Dascalu A., Alexandrescu C., Vancea G., Stana D., Costea D., Zgura A., Serban D., Dumitrescu D., Tudosie M., Serboiu C. (2024). The Relationship between Statin Therapy and Age-Related Macular Degeneration—A Systematic Review. Farmacia.

[14] Fragkiadaki P., Apetroaei M.-M., Kouvidi E., Vakonaki E., Renieri E., Fragkiadoulaki I., Spanakis M., Baliou S., Alegakis A., Tsatsakis A. (2024). The Association between Short Telomere Length and Cardiovascular Disease. Cytogenet. Genome Res..

[15] Tsoukalas D., Buga A., Docea A., Sarandi E., Mitrut R., Renieri E., Spandidos D., Rogoveanu I., Cercelaru L., Niculescu M. (2021). Reversal of Brain Aging by Targeting Telomerase: A Nutraceutical Approach. Int. J. Mol. Med..

[16] Tsatsakis A., Oikonomopoulou T., Nikolouzakis T., Vakonaki E., Tzatzarakis M., Flamourakis M., Renieri E., Fragkiadaki P., Iliaki E., Bachlitzanaki M. (2023). Role of Telomere Length in Human Carcinogenesis (Review). Int. J. Oncol..

[17] Gruber H.-J., Semeraro M.D., Renner W., Herrmann M. (2021). Telomeres and Age-Related Diseases. Biomedicines.

[18] Seitz R. (2016). Human Immunodeficiency Virus (HIV). Transfus. Med. Hemotherapy.

[19] Sever B., Otsuka M., Fujita M., Ciftci H. (2024). A Review of FDA-Approved Anti-HIV-1 Drugs, Anti-Gag Compounds, and Potential Strategies for HIV-1 Eradication. Int. J. Mol. Sci..

[20] Huber A., Baas F.S., van der Ven A.J.A.M., Dos Santos J.C. (2024). Innate Immune Cell Functions Contribute to Spontaneous HIV Control. Curr. HIV/AIDS Rep..

[21] Tekeste S.S., Wilkinson T.A., Weiner E.M., Xu X., Miller J.T., Le Grice S.F.J., Clubb R.T., Chow S.A. (2015). Interaction between Reverse Transcriptase and Integrase Is Required for Reverse Transcription during HIV-1 Replication. J. Virol..

[22] Chou T.C., Maggirwar N.S., Marsden M.D. (2024). HIV Persistence, Latency, and Cure Approaches: Where Are We Now?. Viruses.

[23] Apetroaei M.-M., Velescu B. (2024). Ștefan; Nedea, M.I.; Dinu-Pîrvu, C.E.; Drăgănescu, D.; Fâcă, A.I.; Udeanu, D.I.; Arsene, A.L. The Phenomenon of Antiretroviral Drug Resistance in the Context of Human Immunodeficiency Virus Treatment: Dynamic and Ever Evolving Subject Matter. Biomedicines.

[24] Borrajo A. (2025). Breaking Barriers to an HIV-1 Cure: Innovations in Gene Editing, Immune Modulation, and Reservoir Eradication. Life.

[25] van Schalkwyk C., Mahy M., Johnson L.F., Imai-Eaton J.W. (2024). Updated Data and Methods for the 2023 UNAIDS HIV Estimates. JAIDS J. Acquir. Immune Defic. Syndr..

[26] (2023). UNAIDS Global HIV & AIDS Statistics—Fact Sheet. https://www.unaids.org/sites/default/files/media_asset/UNAIDS_FactSheet_en.pdf.

[27] Hull M.W., Montaner J.S.G. (2013). HIV Treatment as Prevention: The Key to an AIDS-Free Generation. J. Food Drug Anal..

[28] Taramasso L., Andreoni M., Antinori A., Bandera A., Bonfanti P., Bonora S., Borderi M., Castagna A., Cattelan A.M., Celesia B.M. (2023). Pillars of Long-Term Antiretroviral Therapy Success. Pharmacol. Res..

[29] Bertagnolio S., Hermans L., Jordan M.R., Avila-Rios S., Iwuji C., Derache A., Delaporte E., Wensing A., Aves T., Borhan A.S.M. (2021). Clinical Impact of Pretreatment Human Immunodeficiency Virus Drug Resistance in People Initiating Nonnucleoside Reverse Transcriptase Inhibitor–Containing Antiretroviral Therapy: A Systematic Review and Meta-Analysis. J. Infect. Dis..

[30] Chen Z., Wang T., Wu C., Deng K., Wang M., Qi H., Wang N., Li Y., Deng Y., Cao G. (2024). Therapeutic Strategies and Use of Lopinavir/Ritonavir in Patients with Highly Resistant Human Immunodeficiency Virus. Farmacia.

[31] Payagala S., Pozniak A. (2024). The Global Burden of HIV. Clin. Dermatol..

[32] Karris M.Y., Dubé K., Moore A.A. (2020). What Lessons It Might Teach Us? Community Engagement in HIV Research. Curr. Opin. HIV AIDS.

[33] Chahine E.B. (2021). Fostemsavir: The First Oral Attachment Inhibitor for Treatment of HIV-1 Infection. Am. J. Health Pharm..

[34] Beccari M.V., Mogle B.T., Sidman E.F., Mastro K.A., Asiago-Reddy E., Kufel W.D. (2019). Ibalizumab, a Novel Monoclonal Antibody for the Management of Multidrug-Resistant HIV-1 Infection. Antimicrob. Agents Chemother..

[35] Temereanca A., Ruta S. (2023). Strategies to Overcome HIV Drug Resistance-Current and Future Perspectives. Front. Microbiol..

[36] Günthard H.F., Saag M.S., Benson C.A., del Rio C., Eron J.J., Gallant J.E., Hoy J.F., Mugavero M.J., Sax P.E., Thompson M.A. (2016). Antiretroviral Drugs for Treatment and Prevention of HIV Infection in Adults. JAMA.

[37] Lungu G.N., Diaconescu G.I., Dumitrescu F., Docea O.O., Mitrut R., Giubelan L., Zlatian O., Mitrut P. (2024). Liver Damage During Treatment with Reverse-Transcriptase Inhibitors in HIV Patients. Curr. Health Sci. J..

[38] López-Otín C., Blasco M.A., Partridge L., Serrano M., Kroemer G. (2013). The Hallmarks of Aging. Cell.

[39] López-Otín C., Blasco M.A., Partridge L., Serrano M., Kroemer G. (2023). Hallmarks of Aging: An Expanding Universe. Cell.

[40] Tartiere A.G., Freije J.M.P., López-Otín C. (2024). The Hallmarks of Aging as a Conceptual Framework for Health and Longevity Research. Front. Aging.

[41] Biga P.R., Duan J.E., Young T.E., Marks J.R., Bronikowski A., Decena L.P., Randolph E.C., Pavuluri A.G., Li G., Fang Y. (2025). Hallmarks of Aging: A User’s Guide for Comparative Biologists. Ageing Res. Rev..

[42] Premeaux T.A., Ndhlovu L.C. (2023). Decrypting Biological Hallmarks of Aging in People with HIV. Curr. Opin. HIV AIDS.

[43] Montano M., Oursler K.K., Xu K., Sun Y.V., Marconi V.C. (2022). Biological Ageing with HIV Infection: Evaluating the Geroscience Hypothesis. Lancet Health Longev..

[44] Vijg J., Montagna C. (2017). Genome Instability and Aging: Cause or Effect?. Transl. Med. Aging.

[45] Popovic M., Kahl V., Hoch N.C. (2022). Editorial: Genome Instability: Old Problem, New Solutions. Front. Cell Dev. Biol..

[46] Lopez A., Nichols Doyle R., Sandoval C., Nisson K., Yang V., Fregoso O.I. (2022). Viral Modulation of the DNA Damage Response and Innate Immunity: Two Sides of the Same Coin. J. Mol. Biol..

[47] Luftig M.A. (2014). Viruses and the DNA Damage Response: Activation and Antagonism. Annu. Rev. Virol..

[48] Ellwanger J.H., Kulmann-Leal B., Ziliotto M., Chies J.A.B. (2023). HIV Infection, Chromosome Instability, and Micronucleus Formation. Viruses.

[49] Anisenko A., Nefedova A., Agapkina Y., Gottikh M. (2023). Both ATM and DNA-PK Are the Main Regulators of HIV-1 Post-Integrational DNA Repair. Int. J. Mol. Sci..

[50] Mdletshe N., Nel A., Shires K., Mowla S. (2020). HIV Nef Enhances the Expression of Oncogenic C-MYC and Activation-Induced Cytidine Deaminase in Burkitt Lymphoma Cells, Promoting Genomic Instability. Infect. Agent. Cancer.

[51] Robbiani D.F., Bunting S., Feldhahn N., Bothmer A., Camps J., Deroubaix S., McBride K.M., Klein I.A., Stone G., Eisenreich T.R. (2009). AID Produces DNA Double-Strand Breaks in Non-Ig Genes and Mature B Cell Lymphomas with Reciprocal Chromosome Translocations. Mol. Cell.

[52] Poetsch A.R. (2020). The Genomics of Oxidative DNA Damage, Repair, and Resulting Mutagenesis. Comput. Struct. Biotechnol. J..

[53] Isaguliants M., Bayurova E., Avdoshina D., Kondrashova A., Chiodi F., Palefsky J. (2021). Oncogenic Effects of HIV-1 Proteins, Mechanisms Behind. Cancers.

[54] Louboutin J.-P., Agrawal L., Reyes B.A.S., Van Bockstaele E.J., Strayer D.S. (2010). HIV-1 Gp120-Induced Injury to the Blood-Brain Barrier: Role of Metalloproteinases 2 and 9 and Relationship to Oxidative Stress. J. Neuropathol. Exp. Neurol..

[55] Mangino G., Famiglietti M., Capone C., Veroni C., Percario Z.A., Leone S., Fiorucci G., Lülf S., Romeo G., Agresti C. (2015). HIV-1 Myristoylated Nef Treatment of Murine Microglial Cells Activates Inducible Nitric Oxide Synthase, NO2 Production and Neurotoxic Activity. PLoS ONE.

[56] Gutiérrez-Sevilla J.E., Cárdenas-Bedoya J., Escoto-Delgadillo M., Zúñiga-González G.M., Pérez-Ríos A.M., Gómez-Meda B.C., González-Enríquez G.V., Figarola-Centurión I., Chavarría-Avila E., Torres-Mendoza B.M. (2021). Genomic Instability in People Living with HIV. Mutat. Res. Toxicol. Environ. Mutagen..

[57] Lindberg H.K., Wang X., Järventaus H., Falck G.C.-M., Norppa H., Fenech M. (2007). Origin of Nuclear Buds and Micronuclei in Normal and Folate-Deprived Human Lymphocytes. Mutat. Res. Mol. Mech. Mutagen..

[58] Zanet D.L., Thorne A., Singer J., Maan E.J., Sattha B., Le Campion A., Soudeyns H., Pick N., Murray M., Money D.M. (2014). Association between Short Leukocyte Telomere Length and HIV Infection in a Cohort Study: No Evidence of a Relationship with Antiretroviral Therapy. Clin. Infect. Dis..

[59] Gonzalez-Serna A., Ajaykumar A., Gadawski I., Muñoz-Fernández M.A., Hayashi K., Harrigan P.R., Côté H.C.F. (2017). Rapid Decrease in Peripheral Blood Mononucleated Cell Telomere Length After HIV Seroconversion, but Not HCV Seroconversion. J. Acquir. Immune Defic. Syndr..

[60] Pathai S., Lawn S.D., Gilbert C.E., McGuinness D., McGlynn L., Weiss H.A., Port J., Christ T., Barclay K., Wood R. (2013). Accelerated Biological Ageing in HIV-Infected Individuals in South Africa: A Case-Control Study. AIDS.

[61] Yang N.Y., Hsieh A.Y.Y., Chen Z., Campbell A.R., Gadawska I., Kakkar F., Sauve L., Bitnun A., Brophy J., Murray M.C.M. (2024). Chronic and Latent Viral Infections and Leukocyte Telomere Length across the Lifespan of Female and Male Individuals Living with or without HIV. Viruses.

[62] Macamo E.D., Mkhize-Kwitshana Z.L., Mthombeni J., Naidoo P. (2024). The Impact of HIV and Parasite Single Infection and Coinfection on Telomere Length: A Systematic Review. Curr. Issues Mol. Biol..

[63] Macamo E.D., Mkhize-Kwitshana Z.L., Duma Z., Mthombeni J., Naidoo P. (2024). Telomere Length in a South African Population Co-Infected with HIV and Helminths. Curr. Issues Mol. Biol..

[64] Schoepf I.C., Thorball C.W., Ledergerber B., Kootstra N.A., Reiss P., Raffenberg M., Engel T., Braun D.L., Hasse B., Thurnheer C. (2022). Telomere Length Declines in Persons With Human Immunodeficiency Virus Before Antiretroviral Therapy Start but Not After Viral Suppression: A Longitudinal Study Over >17 Years. J. Infect. Dis..

[65] Toljić B., Milašin J., De Luka S.R., Dragović G., Jevtović D., Maslać A., Ristić-Djurović J.L., Trbovich A.M. (2023). HIV-Infected Patients as a Model of Aging. Microbiol. Spectr..

[66] Grosso T.M., Alcamí J., Arribas J.R., Martín M., Sereti I., Tarr P., Cahn P., Clotet B., Sued O., Negredo E. (2022). HIV and Aging, Biological Mechanisms, and Therapies: What Do We Know?. Aids Rev..

[67] Mehta S.R., Iudicello J.E., Lin J., Ellis R.J., Morgan E., Okwuegbuna O., Cookson D., Karris M., Saloner R., Heaton R. (2021). Telomere Length Is Associated with HIV Infection, Methamphetamine Use, Inflammation, and Comorbid Disease Risk. Drug Alcohol Depend..

[68] Schoepf I.C., Thorball C.W., Ledergerber B., Engel T., Raffenberg M., Kootstra N.A., Reiss P., Hasse B., Marzolini C., Thurnheer C. (2021). Coronary Artery Disease-Associated and Longevity-Associated Polygenic Risk Scores for Prediction of Coronary Artery Disease Events in Persons Living With Human Immunodeficiency Virus: The Swiss HIV Cohort Study. Clin. Infect. Dis..

[69] Kurashova N.A., Vanyarkina A.S., Petrova A.G., Rychkova L.V., Kolesnikov S.I., Darenskaya M.A., Moskaleva E.V., Kolesnikova L.I. (2023). Length of Leukocyte Telomeres in Newborns of HIV-Infected Mothers. Bull. Exp. Biol. Med..

[70] Lu A.T., Seeboth A., Tsai P.-C., Sun D., Quach A., Reiner A.P., Kooperberg C., Ferrucci L., Hou L., Baccarelli A.A. (2019). DNA Methylation-Based Estimator of Telomere Length. Aging.

[71] Liang X., Aouizerat B.E., So-Armah K., Cohen M.H., Marconi V.C., Xu K., Justice A.C. (2024). DNA Methylation-Based Telomere Length Is Associated with HIV Infection, Physical Frailty, Cancer, and All-Cause Mortality. Aging Cell.

[72] Hu H., Li B., Duan S. (2018). The Alteration of Subtelomeric DNA Methylation in Aging-Related Diseases. Front. Genet..

[73] Mender I., Shay J.W. (2015). Telomere Restriction Fragment (TRF) Analysis. Bio-Protocol.

[74] Pearce E.E., Horvath S., Katta S., Dagnall C., Aubert G., Hicks B.D., Spellman S.R., Katki H., Savage S.A., Alsaggaf R. (2021). DNA-Methylation-Based Telomere Length Estimator: Comparisons with Measurements from Flow FISH and QPCR. Aging.

[75] Handy D.E., Castro R., Loscalzo J. (2011). Epigenetic Modifications. Circulation.

[76] Titanji B.K., Gwinn M., Marconi V.C., Sun Y.V. (2022). Epigenome-Wide Epidemiologic Studies of Human Immunodeficiency Virus Infection, Treatment, and Disease Progression. Clin. Epigenetics.

[77] Lange U.C., Verdikt R., Ait-Ammar A., Van Lint C. (2020). Epigenetic Crosstalk in Chronic Infection with HIV-1. Semin. Immunopathol..

[78] Arumugam T., Ramphal U., Adimulam T., Chinniah R., Ramsuran V. (2021). Deciphering DNA Methylation in HIV Infection. Front. Immunol..

[79] Nunes J.M., Furtado M.N., de Morais Nunes E.R., Sucupira M.C.A., Diaz R.S., Janini L.M.R. (2018). Modulation of Epigenetic Factors during the Early Stages of HIV-1 Infection in CD4+ T Cells in Vitro. Virology.

[80] Abdel-Hameed E.A., Ji H., Sherman K.E., Shata M.T.M. (2014). Epigenetic Modification of FOXP3 in Patients With Chronic HIV Infection. JAIDS J. Acquir. Immune Defic. Syndr..

[81] Pion M., Jaramillo-Ruiz D., Martínez A., Muñoz-Fernández M.A., Correa-Rocha R. (2013). HIV Infection of Human Regulatory T Cells Downregulates Foxp3 Expression by Increasing DNMT3b Levels and DNA Methylation in the FOXP3 Gene. AIDS.

[82] Abdel-Hameed E.A., Ji H., Shata M.T. (2016). HIV-Induced Epigenetic Alterations in Host Cells. Adv. Exp. Med. Biol..

[83] Sadowski I., Hashemi F.B. (2019). Strategies to Eradicate HIV from Infected Patients: Elimination of Latent Provirus Reservoirs. Cell. Mol. Life Sci..

[84] Battistini A., Sgarbanti M. (2014). HIV-1 Latency: An Update of Molecular Mechanisms and Therapeutic Strategies. Viruses.

[85] Ma X., Chen T., Peng Z., Wang Z., Liu J., Yang T., Wu L., Liu G., Zhou M., Tong M. (2021). Histone Chaperone CAF-1 Promotes HIV-1 Latency by Leading the Formation of Phase-separated Suppressive Nuclear Bodies. EMBO J..

[86] Marzio G., Tyagi M., Gutierrez M.I., Giacca M. (1998). HIV-1 Tat Transactivator Recruits P300 and CREB-Binding Protein Histone Acetyltransferases to the Viral Promoter. Proc. Natl. Acad. Sci. USA.

[87] Margolis D.M. (2011). Histone Deacetylase Inhibitors and HIV Latency. Curr. Opin. HIV AIDS.

[88] Lenasi T., Contreras X., Peterlin B.M. (2008). Transcriptional Interference Antagonizes Proviral Gene Expression to Promote HIV Latency. Cell Host Microbe.

[89] Espíndola M.S., Soares L.S., Galvão-Lima L.J., Zambuzi F.A., Cacemiro M.C., Brauer V.S., Marzocchi-Machado C.M., de Souza Gomes M., Amaral L.R., Martins-Filho O.A. (2018). Epigenetic Alterations Are Associated with Monocyte Immune Dysfunctions in HIV-1 Infection. Sci. Rep..

[90] Labbadia J., Morimoto R.I. (2015). The Biology of Proteostasis in Aging and Disease. Annu. Rev. Biochem..

[91] Mainolfi N., Rasmusson T. (2017). Targeted Protein Degradation. Annu. Rep. Med. Chem..

[92] Seissler T., Marquet R., Paillart J.-C. (2017). Hijacking of the Ubiquitin/Proteasome Pathway by the HIV Auxiliary Proteins. Viruses.

[93] Iwatani Y., Chan D.S.B., Liu L., Yoshii H., Shibata J., Yamamoto N., Levin J.G., Gronenborn A.M., Sugiura W. (2009). HIV-1 Vif-Mediated Ubiquitination/Degradation of APOBEC3G Involves Four Critical Lysine Residues in Its C-Terminal Domain. Proc. Natl. Acad. Sci. USA.

[94] Li Y.-L., Langley C.A., Azumaya C.M., Echeverria I., Chesarino N.M., Emerman M., Cheng Y., Gross J.D. (2023). The Structural Basis for HIV-1 Vif Antagonism of Human APOBEC3G. Nature.

[95] Ballana E., Badia R., Terradas G., Torres-Torronteras J., Ruiz A., Pauls E., Riveira-Muñoz E., Clotet B., Martí R., Esté J.A. (2014). SAMHD1 Specifically Affects the Antiviral Potency of Thymidine Analog HIV Reverse Transcriptase Inhibitors. Antimicrob. Agents Chemother..

[96] Zheng Y.-H., Jeang K.-T., Tokunaga K. (2012). Host Restriction Factors in Retroviral Infection: Promises in Virus-Host Interaction. Retrovirology.

[97] Hofmann H., Logue E.C., Bloch N., Daddacha W., Polsky S.B., Schultz M.L., Kim B., Landau N.R. (2012). The Vpx Lentiviral Accessory Protein Targets SAMHD1 for Degradation in the Nucleus. J. Virol..

[98] Dubé M., Paquay C., Roy B.B., Bego M.G., Mercier J., Cohen É.A. (2011). HIV-1 Vpu Antagonizes BST-2 by Interfering Mainly with the Trafficking of Newly Synthesized BST-2 to the Cell Surface. Traffic.

[99] Lata S., Mishra R., Banerjea A.C. (2018). Proteasomal Degradation Machinery: Favorite Target of HIV-1 Proteins. Front. Microbiol..

[100] Remoli A.L., Marsili G., Perrotti E., Acchioni C., Sgarbanti M., Borsetti A., Hiscott J., Battistini A. (2016). HIV-1 Tat Recruits HDM2 E3 Ligase To Target IRF-1 for Ubiquitination and Proteasomal Degradation. mBio.

[101] Gómez-Virgilio L., Silva-Lucero M.-C., Flores-Morelos D.-S., Gallardo-Nieto J., Lopez-Toledo G., Abarca-Fernandez A.-M., Zacapala-Gómez A.-E., Luna-Muñoz J., Montiel-Sosa F., Soto-Rojas L.O. (2022). Autophagy: A Key Regulator of Homeostasis and Disease: An Overview of Molecular Mechanisms and Modulators. Cells.

[102] Feng Y., He D., Yao Z., Klionsky D.J. (2014). The Machinery of Macroautophagy. Cell Res..

[103] Nieto-Torres J.L., Hansen M. (2021). Macroautophagy and Aging: The Impact of Cellular Recycling on Health and Longevity. Mol. Aspects Med..

[104] Cheney L., Guzik H., Macaluso F.P., Macian F., Cuervo A.M., Berman J.W. (2020). HIV Nef and Antiretroviral Therapy Have an Inhibitory Effect on Autophagy in Human Astrocytes That May Contribute to HIV-Associated Neurocognitive Disorders. Cells.

[105] Castro-Gonzalez S., Shi Y., Colomer-Lluch M., Song Y., Mowery K., Almodovar S., Bansal A., Kirchhoff F., Sparrer K., Liang C. (2021). HIV-1 Nef Counteracts Autophagy Restriction by Enhancing the Association between BECN1 and Its Inhibitor BCL2 in a PRKN-Dependent Manner. Autophagy.

[106] Sun Y., Xu M., Duan Q., Bryant J.L., Xu X. (2024). The Role of Autophagy in the Progression of HIV Infected Cardiomyopathy. Front. Cell Dev. Biol..

[107] Di Micco R., Krizhanovsky V., Baker D., d’Adda di Fagagna F. (2021). Cellular Senescence in Ageing: From Mechanisms to Therapeutic Opportunities. Nat. Rev. Mol. Cell Biol..

[108] Ernst P., Heidel F.H. (2021). Molecular Mechanisms of Senescence and Implications for the Treatment of Myeloid Malignancies. Cancers.

[109] Jin P., Duan X., Li L., Zhou P., Zou C., Xie K. (2024). Cellular Senescence in Cancer: Molecular Mechanisms and Therapeutic Targets. Med. Comm..

[110] Sheekey E., Narita M. (2023). P53 in Senescence—It’s a Marathon, Not a Sprint. FEBS J..

[111] Gao M., Li H., Zhang J. (2025). RB Functions as a Key Regulator of Senescence and Tumor Suppression. Semin. Cancer Biol..

[112] Talluri S., Dick F.A. (2012). Regulation of Transcription and Chromatin Structure by PRB: Here, There and Everywhere. Cell Cycle.

[113] Liu Z., Liang Q., Ren Y., Guo C., Ge X., Wang L., Cheng Q., Luo P., Zhang Y., Han X. (2023). Immunosenescence: Molecular Mechanisms and Diseases. Signal Transduct. Target. Ther..

[114] Desai S., Landay A. (2010). Early Immune Senescence in HIV Disease. Curr. HIV/AIDS Rep..

[115] Lara-Aguilar V., Llamas-Adán M., Brochado-Kith Ó., Crespo-Bermejo C., Grande-García S., Arca-Lafuente S., de los Santos I., Prado C., Alía M., Sainz-Pinós C. (2024). Low-Level HIV-1 Viremia Affects T-Cell Activation and Senescence in Long-Term Treated Adults in the INSTI Era. J. Biomed. Sci..

[116] Pangrazzi L., Meryk A. (2024). Molecular and Cellular Mechanisms of Immunosenescence: Modulation Through Interventions and Lifestyle Changes. Biology.

[117] Soares L.S., Espíndola M.S., Zambuzi F.A., Galvão-Lima L.J., Cacemiro M.C., Soares M.R., Santana B.A., Calado R.T., Bollela V.R., Frantz F.G. (2020). Immunosenescence in Chronic HIV Infected Patients Impairs Essential Functions of Their Natural Killer Cells. Int. Immunopharmacol..

[118] Deeks S.G., Verdin E., McCune J.M. (2012). Immunosenescence and HIV. Curr. Opin. Immunol..

[119] Ivanov A.V., Valuev-Elliston V.T., Ivanova O.N., Kochetkov S.N., Starodubova E.S., Bartosch B., Isaguliants M.G. (2016). Oxidative Stress during HIV Infection: Mechanisms and Consequences. Oxid. Med. Cell. Longev..

[120] Fouquerel E., Barnes R.P., Uttam S., Watkins S.C., Bruchez M.P., Opresko P.L. (2019). Targeted and Persistent 8-Oxoguanine Base Damage at Telomeres Promotes Telomere Loss and Crisis. Mol. Cell.

[121] Apetroaei M.-M., Fragkiadaki P., Velescu B. (2024). Ștefan; Baliou, S.; Renieri, E.; Dinu-Pirvu, C.E.; Drăgănescu, D.; Vlăsceanu, A.M.; Nedea, M.I. (Ilie); Udeanu, D.I.; et al. Pharmacotherapeutic Considerations on Telomere Biology: The Positive Effect of Pharmacologically Active Substances on Telomere Length. Int. J. Mol. Sci..

[122] Wang J., Li C., Han J., Xue Y., Zheng X., Wang R., Radak Z., Nakabeppu Y., Boldogh I., Ba X. (2025). Reassessing the Roles of Oxidative DNA Base Lesion 8-OxoGua and Repair Enzyme OGG1 in Tumorigenesis. J. Biomed. Sci..

[123] Barnes R.P., Fouquerel E., Opresko P.L. (2019). The Impact of Oxidative DNA Damage and Stress on Telomere Homeostasis. Mech. Ageing Dev..

[124] Harshithkumar R., Shah P., Jadaun P., Mukherjee A. (2024). ROS Chronicles in HIV Infection: Genesis of Oxidative Stress, Associated Pathologies, and Therapeutic Strategies. Curr. Issues Mol. Biol..

[125] Figarola-Centurión I., Escoto-Delgadillo M., González-Enríquez G.V., Gutiérrez-Sevilla J.E., Vázquez-Valls E., Torres-Mendoza B.M. (2022). Sirtuins Modulation: A Promising Strategy for HIV-Associated Neurocognitive Impairments. Int. J. Mol. Sci..

[126] Xiao M., Zhong H., Xia L., Tao Y., Yin H. (2017). Pathophysiology of Mitochondrial Lipid Oxidation: Role of 4-Hydroxynonenal (4-HNE) and Other Bioactive Lipids in Mitochondria. Free Radic. Biol. Med..

[127] Kowalczyk P., Sulejczak D., Kleczkowska P., Bukowska-Ośko I., Kucia M., Popiel M., Wietrak E., Kramkowski K., Wrzosek K., Kaczyńska K. (2021). Mitochondrial Oxidative Stress—A Causative Factor and Therapeutic Target in Many Diseases. Int. J. Mol. Sci..

[128] Schank M., Zhao J., Moorman J.P., Yao Z.Q. (2021). The Impact of HIV- and ART-Induced Mitochondrial Dysfunction in Cellular Senescence and Aging. Cells.

[129] Rodriguez N.R., Fortune T., Hegde E., Weinstein M.P., Keane A.M., Mangold J.F., Swartz T.H. (2024). Oxidative Phosphorylation in HIV-1 Infection: Impacts on Cellular Metabolism and Immune Function. Front. Immunol..

[130] Ostermann P.N., Evering T.H. (2024). The Impact of Aging on HIV-1-Related Neurocognitive Impairment. Ageing Res. Rev..

[131] Crater J.M., Nixon D.F., Furler O’Brien R.L. (2022). HIV-1 Replication and Latency Are Balanced by MTOR-Driven Cell Metabolism. Front. Cell. Infect. Microbiol..

[132] Akbay B., Shmakova A., Vassetzky Y., Dokudovskaya S. (2020). Modulation of MTORC1 Signaling Pathway by HIV-1. Cells.

[133] Saxton R.A., Sabatini D.M. (2017). MTOR Signaling in Growth, Metabolism, and Disease. Cell.

[134] Willig A.L., Overton E.T. (2016). Metabolic Complications and Glucose Metabolism in HIV Infection: A Review of the Evidence. Curr. HIV/AIDS Rep..

[135] Houtkooper R.H., Pirinen E., Auwerx J. (2012). Sirtuins as Regulators of Metabolism and Healthspan. Nat. Rev. Mol. Cell Biol..

[136] Bhutta M., Gallo E., Borenstein R. (2021). Multifaceted Role of AMPK in Viral Infections. Cells.

[137] Hardie D.G., Ross F.A., Hawley S.A. (2012). AMPK: A Nutrient and Energy Sensor That Maintains Energy Homeostasis. Nat. Rev. Mol. Cell Biol..

[138] Lawton A., Tripodi N., Feehan J. (2024). Running on Empty: Exploring Stem Cell Exhaustion in Geriatric Musculoskeletal Disease. Maturitas.

[139] Herd C.L., Mellet J., Mashingaidze T., Durandt C., Pepper M.S. (2023). Consequences of HIV Infection in the Bone Marrow Niche. Front. Immunol..

[140] Bogeska R., Mikecin A.-M., Kaschutnig P., Fawaz M., Büchler-Schäff M., Le D., Ganuza M., Vollmer A., Paffenholz S.V., Asada N. (2022). Inflammatory Exposure Drives Long-Lived Impairment of Hematopoietic Stem Cell Self-Renewal Activity and Accelerated Aging. Cell Stem Cell.

[141] Zhao H.G., Deininger M.W. (2023). Always stressed but never exhausted: How stem cells in myeloid neoplasms avoid extinction in inflammatory conditions. Blood.

[142] Abbas W., Herbein G. (2013). T-Cell Signaling in HIV-1 Infection. Open Virol. J..

[143] Herbein G., Gras G., Khan K.A., Abbas W. (2010). Macrophage Signaling in HIV-1 Infection. Retrovirology.

[144] Liu R., Lin Y., Jia R., Geng Y., Liang C., Tan J., Qiao W. (2014). HIV-1 Vpr Stimulates NF-ΚB and AP-1 Signaling by Activating TAK1. Retrovirology.

[145] Fiume G., Vecchio E., De Laurentiis A., Trimboli F., Palmieri C., Pisano A., Falcone C., Pontoriero M., Rossi A., Scialdone A. (2012). Human Immunodeficiency Virus-1 Tat Activates NF-ΚB via Physical Interaction with IκB-α and P65. Nucleic Acids Res..

[146] Rahman M.M., McFadden G. (2011). Modulation of NF-ΚB Signalling by Microbial Pathogens. Nat. Rev. Microbiol..

[147] Cafaro A., Schietroma I., Sernicola L., Belli R., Campagna M., Mancini F., Farcomeni S., Pavone-Cossut M.R., Borsetti A., Monini P. (2024). Role of HIV-1 Tat Protein Interactions with Host Receptors in HIV Infection and Pathogenesis. Int. J. Mol. Sci..

[148] Horie R., Ishida T., Maruyama-Nagai M., Ito K., Watanabe M., Yoneyama A., Higashihara M., Kimura S., Watanabe T. (2007). TRAF Activation of C/EBPβ (NF-IL6) via P38 MAPK Induces HIV-1 Gene Expression in Monocytes/Macrophages. Microbes Infect..

[149] Muthumani K., Choo A.Y., Shedlock D.J., Laddy D.J., Sundaram S.G., Hirao L., Wu L., Thieu K.P., Chung C.W., Lankaraman K.M. (2008). Human Immunodeficiency Virus Type 1 Nef Induces Programmed Death 1 Expression through a P38 Mitogen-Activated Protein Kinase-Dependent Mechanism. J. Virol..

[150] Jacob R.A., Johnson A.L., Pawlak E.N., Dirk B.S., Van Nynatten L.R., Haeryfar S.M.M., Dikeakos J.D. (2017). The Interaction between HIV-1 Nef and Adaptor Protein-2 Reduces Nef-Mediated CD4+ T Cell Apoptosis. Virology.

[151] Pasquereau S., Herbein G. (2022). CounterAKTing HIV: Toward a “Block and Clear” Strategy?. Front. Cell. Infect. Microbiol..

[152] Markle T.J., Mwimanzi P., Brockman M.A. (2013). HIV-1 Nef and T-Cell Activation: A History of Contradictions. Future Virol..

[153] Cho H.J., Kuo A.M.-S., Bertrand L., Toborek M. (2017). HIV Alters Gap Junction-Mediated Intercellular Communication in Human Brain Pericytes. Front. Mol. Neurosci..

[154] Bakhanashvili M. (2024). The Role of Tumor Suppressor P53 Protein in HIV–Host Cell Interactions. Cells.

[155] Periyasamy P., Thangaraj A., Bendi V.S., Buch S. (2019). HIV-1 Tat-Mediated Microglial Inflammation Involves a Novel MiRNA-34a-NLRC5-NFκB Signaling Axis. Brain Behav. Immun..

[156] Bartz S.R., Emerman M. (1999). Human Immunodeficiency Virus Type 1 Tat Induces Apoptosis and Increases Sensitivity to Apoptotic Signals by Up-Regulating FLICE/Caspase-8. J. Virol..

[157] Conti L., Rainaldi G., Matarrese P., Varano B., Rivabene R., Columba S., Sato A., Belardelli F., Malorni W., Gessani S. (1998). The HIV-1 Vpr Protein Acts as a Negative Regulator of Apoptosis in a Human Lymphoblastoid T Cell Line: Possible Implications for the Pathogenesis of AIDS. J. Exp. Med..

[158] Verma S., Ali A., Arora S., Banerjea A.C. (2011). Inhibition of β-TrcP–Dependent Ubiquitination of P53 by HIV-1 Vpu Promotes P53–Mediated Apoptosis in Human T Cells. Blood.

[159] Hu Q., Bian Q., Rong D., Wang L., Song J., Huang H.-S., Zeng J., Mei J., Wang P.-Y. (2023). JAK/STAT Pathway: Extracellular Signals, Diseases, Immunity, and Therapeutic Regimens. Front. Bioeng. Biotechnol..

[160] Wang L., Yukselten Y., Nuwagaba J., Sutton R.E. (2024). JAK/STAT Signaling Pathway Affects CCR5 Expression in Human CD4 + T Cells. Sci. Adv..

[161] Gargan S., Ahmed S., Mahony R., Bannan C., Napoletano S., O’Farrelly C., Borrow P., Bergin C., Stevenson N.J. (2018). HIV-1 Promotes the Degradation of Components of the Type 1 IFN JAK/STAT Pathway and Blocks Anti-Viral ISG Induction. eBioMedicine.

[162] Fan Y., Timani K., He J. (2015). STAT3 and Its Phosphorylation Are Involved in HIV-1 Tat-Induced Transactivation of Glial Fibrillary Acidic Protein. Curr. HIV Res..

[163] Nguyen N.V., Tran J.T., Sanchez D.J. (2018). HIV Blocks Type I IFN Signaling through Disruption of STAT1 Phosphorylation. Innate Immun..

[164] Perdaens O., van Pesch V. (2022). Molecular Mechanisms of Immunosenescene and Inflammaging: Relevance to the Immunopathogenesis and Treatment of Multiple Sclerosis. Front. Neurol..

[165] Ajoolabady A., Pratico D., Tang D., Zhou S., Franceschi C., Ren J. (2024). Immunosenescence and Inflammaging: Mechanisms and Role in Diseases. Ageing Res. Rev..

[166] Min A.K., Fortune T., Rodriguez N., Hedge E., Swartz T.H. (2023). Inflammasomes as Mediators of Inflammation in HIV-1 Infection. Transl. Res..

[167] Zevin A.S., McKinnon L., Burgener A., Klatt N.R. (2016). Microbial Translocation and Microbiome Dysbiosis in HIV-Associated Immune Activation. Curr. Opin. HIV AIDS.

[168] Gogokhia L., Taur Y., Juluru K., Yagan N., Zhu Y.-S., Pamer E., Glesby M.J. (2020). Intestinal Dysbiosis and Markers of Systemic Inflammation in Viscerally and Generally Obese Persons Living With HIV. JAIDS J. Acquir. Immune Defic. Syndr..

[169] Fulcher J.A., Li F., Tobin N.H., Zabih S., Elliott J., Clark J.L., D’Aquila R., Mustanski B., Kipke M.D., Shoptaw S. (2022). Gut Dysbiosis and Inflammatory Blood Markers Precede HIV with Limited Changes after Early Seroconversion. eBioMedicine.

[170] Zaongo S.D., Ouyang J., Isnard S., Zhou X., Harypursat V., Cui H., Routy J.-P., Chen Y. (2023). Candida Albicans Can Foster Gut Dysbiosis and Systemic Inflammation during HIV Infection. Gut Microbes.

[171] Martín-Escolano R., Virseda-Berdices A., Berenguer J., González-García J., Brochado-Kith O., Fernández-Rodríguez A., Díez C., Hontañon V., Resino S., Jiménez-Sousa M.Á. (2024). Immune Checkpoint Proteins Are Associated with Persistently High Liver Stiffness after Successful HCV Treatment in People with HIV: A Retrospective Study. Front. Immunol..

[172] Porichis F., Kaufmann D.E. (2012). Role of PD-1 in HIV Pathogenesis and as Target for Therapy. Curr. HIV/AIDS Rep..

[173] Baroncini L., Muller C.K.S., Kadzioch N.P., Wolfensberger R., Russenberger D., Bredl S., Mlambo T., Speck R.F. (2024). Pro-Inflammatory Macrophages Suppress HIV Replication in Humanized Mice and Ex Vivo Co-Cultures. Front. Immunol..

[174] Khansari N., Shakiba Y., Mahmoudi M. (2009). Chronic Inflammation and Oxidative Stress as a Major Cause of Age- Related Diseases and Cancer. Recent Pat. Inflamm. Allergy Drug Discov..

[175] Nousis L., Kanavaros P., Barbouti A. (2023). Oxidative Stress-Induced Cellular Senescence: Is Labile Iron the Connecting Link?. Antioxidants.

[176] de Magalhães J.P., Passos J.F. (2018). Stress, Cell Senescence and Organismal Ageing. Mech. Ageing Dev..

[177] Ishizaka A., Koga M., Mizutani T., Parbie P.K., Prawisuda D., Yusa N., Sedohara A., Kikuchi T., Ikeuchi K., Adachi E. (2021). Unique Gut Microbiome in HIV Patients on Antiretroviral Therapy (ART) Suggests Association with Chronic Inflammation. Microbiol. Spectr..

[178] Russo E., Nannini G., Sterrantino G., Kiros S.T., Di Pilato V., Coppi M., Baldi S., Niccolai E., Ricci F., Ramazzotti M. (2022). Effects of Viremia and CD4 Recovery on Gut “Microbiome-Immunity” Axis in Treatment-Naïve HIV-1-Infected Patients Undergoing Antiretroviral Therapy. World J. Gastroenterol..

[179] Xie Y., Sun J., Wei L., Jiang H., Hu C., Yang J., Huang Y., Ruan B., Zhu B. (2021). Altered Gut Microbiota Correlate with Different Immune Responses to HAART in HIV-Infected Individuals. BMC Microbiol..

[180] Ray S., Narayanan A., Giske C.G., Neogi U., Sönnerborg A., Nowak P. (2021). Altered Gut Microbiome under Antiretroviral Therapy: Impact of Efavirenz and Zidovudine. ACS Infect. Dis..

[181] Pérez-Santiago J., Marquine M.J., Cookson D., Giraud-Colón R., Heaton R.K., Grant I., Ellis R.J., Letendre S.L., Peterson S.N. (2021). Gut Microbiota Dysbiosis Is Associated with Worse Emotional States in HIV Infection. J. Neurovirol..

[182] Brenchley J.M., Serrano-Villar S. (2024). From Dysbiosis to Defense: Harnessing the Gut Microbiome in HIV/SIV Therapy. Microbiome.

[183] Montejano R., Stella-Ascariz N., Monge S., Bernardino J.I., Pérez-Valero I., Montes M.L., Valencia E., Martín-Carbonero L., Moreno V., González-García J. (2017). Impact of Antiretroviral Treatment Containing Tenofovir Difumarate on the Telomere Length of Aviremic HIV-Infected Patients. JAIDS J. Acquir. Immune Defic. Syndr..

[184] Wang Y., Brummel S.S., Beilstein-Wedel E., Dagnall C.L., Hazra R., Kacanek D., Chadwick E.G., Marsit C.J., Chanock S.J., Savage S.A. (2019). Association between Zidovudine-Containing Antiretroviral Therapy Exposure in Utero and Leukocyte Telomere Length at Birth. AIDS.

[185] Montejano R., Stella-Ascariz N., Monge S., Bernardino J.I., Pérez-Valero I., Montes M.L., Valencia E., Martín-Carbonero L., Moreno V., González-Garcia J. (2018). Impact of Nucleos(t)Ide Reverse Transcriptase Inhibitors on Blood Telomere Length Changes in a Prospective Cohort of Aviremic HIV–Infected Adults. J. Infect. Dis..

[186] Rodríguez-Centeno J., Esteban-Cantos A., Montejano R., Stella-Ascariz N., De Miguel R., Mena-Garay B., Saiz-Medrano G., Alejos B., Jiménez-González M., Bernardino J.I. (2022). Effects of Tenofovir on Telomeres, Telomerase and T Cell Maturational Subset Distribution in Long-Term Aviraemic HIV-Infected Adults. J. Antimicrob. Chemother..

[187] Bollmann F.M. (2013). Telomerase Inhibition May Contribute to Accelerated Mitochondrial Aging Induced by Anti-Retroviral HIV Treatment. Med. Hypotheses.

[188] Hukezalie K.R., Thumati N.R., Côté H.C.F., Wong J.M.Y. (2012). In Vitro and Ex Vivo Inhibition of Human Telomerase by Anti-HIV Nucleoside Reverse Transcriptase Inhibitors (NRTIs) but Not by Non-NRTIs. PLoS ONE.

[189] Torres R.A., Lewis W. (2014). Aging and HIV/AIDS: Pathogenetic Role of Therapeutic Side Effects. Lab. Investig..

[190] Leeansyah E., Cameron P.U., Solomon A., Tennakoon S., Velayudham P., Gouillou M., Spelman T., Hearps A., Fairley C., Smit D.V. (2013). Inhibition of Telomerase Activity by Human Immunodeficiency Virus (HIV) Nucleos(t)Ide Reverse Transcriptase Inhibitors: A Potential Factor Contributing to HIV-Associated Accelerated Aging. J. Infect. Dis..

[191] Bukic E., Milasin J., Toljic B., Jadzic J., Jevtovic D., Obradovic B., Dragovic G. (2023). Association between Combination Antiretroviral Therapy and Telomere Length in People Living with Human Immunodeficiency Virus. Biology.

[192] Rusu R., Ababei D., Macadan I., Ciobîcă A., Paraschiv M., Bild W., Moraru A., Nicolae C., Bild V. (2023). Factors That Influence Treatment Adherence–Realities, Controversies, Perspectives. Farmacia.

[193] Deeks S.G. (2011). HIV Infection, Inflammation, Immunosenescence, and Aging. Annu. Rev. Med..

[194] Darcis G., Moutschen M. (2017). The Effect of Treatment Simplification on HIV Reservoirs. Lancet HIV.

[195] Laiho M., Saksela O., Andreasen P.A., Keski-Oja J. (1986). Enhanced Production and Extracellular Deposition of the Endothelial-Type Plasminogen Activator Inhibitor in Cultured Human Lung Fibroblasts by Transforming Growth Factor-Beta. J. Cell Biol..

[196] Côté H.C.F., Soudeyns H., Thorne A., Alimenti A., Lamarre V., Maan E.J., Sattha B., Singer J., Lapointe N., Money D.M. (2012). Leukocyte Telomere Length in HIV-Infected and HIV-Exposed Uninfected Children: Shorter Telomeres with Uncontrolled HIV Viremia. PLoS ONE.

[197] Minami R., Takahama S., Yamamoto M. (2019). Correlates of Telomere Length Shortening in Peripheral Leukocytes of HIV-Infected Individuals and Association with Leukoaraiosis. PLoS ONE.

[198] Lombardi F., Sanfilippo A., Fabbiani M., Borghetti A., Ciccullo A., Tamburrini E., Di Giambenedetto S. (2023). Blood Telomere Length Gain in People Living with HIV Switching to Dolutegravir plus Lamivudine versus Continuing Triple Regimen: A Longitudinal, Prospective, Matched, Controlled Study. J. Antimicrob. Chemother..

[199] Molina-Carrión S., Brochado-Kith Ó., González-García J., Berenguer J., Díez C., Llop E., Hontañón V., Ibañez-Samaniego L., Montes M.L., Resino S. (2020). Telomere Length Increase in HIV/HCV-Coinfected Patients with Cirrhosis after HCV Eradication with Direct-Acting Antivirals. J. Clin. Med..

[200] Monnin A., Vizeneux A., Nagot N., Eymard-Duvernay S., Meda N., Singata-Madliki M., Ndeezi G., Tumwine J.K., Kankasa C., Goga A. (2021). Longitudinal Follow-Up of Blood Telomere Length in HIV-Exposed Uninfected Children Having Received One Year of Lopinavir/Ritonavir or Lamivudine as Prophylaxis. Children.

[201] Chalouni M., Rodriguez-Centeno J., Samri A., Blanco J., Stella-Ascariz N., Wallet C., Knobel H., Zucman D., Alejos Ferreras B., Autran B. (2020). Correlation between Blood Telomere Length and CD4+ CD8+ T-Cell Subsets Changes 96 Weeks after Initiation of Antiretroviral Therapy in HIV-1–Positive Individuals. PLoS ONE.

[202] Saberi S., Kalloger S.E., Zhu M.M.T., Sattha B., Maan E.J., van Schalkwyk J., Money D.M., Côté H.C.F. (2019). Dynamics of Leukocyte Telomere Length in Pregnant Women Living with HIV, and HIV-Negative Pregnant Women: A Longitudinal Observational Study. PLoS ONE.

[203] Quiros-Roldan E., Properzi M., Paghera S., Raffetti E., Castelli F., Imberti L. (2020). Factors Associated with Immunosenescence during Early Adulthood in HIV-Infected Patients after Durable Efficient Combination Antiretroviral Therapy. Sci. Rep..

